# Iron Deficiency: Global Trends and Projections from 1990 to 2050

**DOI:** 10.3390/nu16203434

**Published:** 2024-10-10

**Authors:** Li Wang, Dan Liang, Hengqian Huangfu, Xinfu Shi, Shuang Liu, Panpan Zhong, Zhen Luo, Changwen Ke, Yingsi Lai

**Affiliations:** 1 Department of Medical Statistics, School of Public Health, Sun Yat-sen University, Guangzhou 510080, China; wangli237@mail2.sysu.edu.cn (L.W.); huangfhq@mail2.sysu.edu.cn (H.H.); shixf6@mail2.sysu.edu.cn (X.S.); 2 Department of Immunology and Microbiology, College of Life Science and Technology, Jinan University, Guangzhou 510632, China; liangdancat@stu2022.jnu.edu.cn (D.L.); liushuang@stu.jnu.edu.cn (S.L.); zpp2819195@stu.jnu.edu.cn (P.Z.); 3 Guangdong Provincial Key Laboratory for Emergency Detection and Research on Pathogen of Emerging Infectious Disease, Guangdong Provincial Center for Disease Control and Prevention, Guangdong Workstation for Emerging Infectious Disease Control and Prevention, Guangzhou 511430, China; 4 Sun Yat-sen Global Health Institute, Sun Yat-sen University, Guangzhou 510080, China; 5 Research Center of Health Informatics, Sun Yat-sen University, Guangzhou 510080, China; 6 Guangdong Key Laboratory of Health Informatics, Guangzhou 510080, China; 7 Guangzhou Joint Research Center for Disease Surveillance, Early Warning, and Risk Assessment, Guangzhou 510080, China

**Keywords:** iron deficiency, global burden of disease, SHAP, XGBoost, forecast

## Abstract

Background: Iron deficiency (ID) remains the leading cause of anemia, affects a vast number of persons globally, and continues to be a significant global health burden. Comprehending the patterns of ID burden is essential for developing targeted public health policies. Methods: Using data from the Global Burden of Disease (GBD) 2021 study for the years 1990–2021, the XGBoost model was constructed to predict prevalence and disability-adjusted life years (DALYs) for the period 2022–2050, based on key demographic variables. Shapley Additive exPlanations (SHAP) values were applied to interpret the contributions of each variable to the model’s predictions. Additionally, the Age–Period–Cohort (APC) model was used to evaluate the effects of age, period, and birth cohort on both prevalence and DALYs. The relationship between the Socio-Demographic Index (SDI) and ID’s age-standardized prevalence rate (ASPR) as well as the age-standardized DALYs rate (ASDR) was also analyzed to assess the influence of socioeconomic development on disease burden. Results: The global prevalent cases of ID grew from 984.61 million in 1990 to 1270.64 million in 2021 and are projected to reach 1439.99 million by 2050. Similarly, global DALYs from ID increased from 28.41 million in 1990 to 32.32 million in 2021, with a projected rise to 36.13 million by 2050. The ASPR declined from 18,204/100,000 in 1990 to 16,433/100,000 in 2021, with an estimated annual percentage change (EAPC) of −0.36% over this period. It is expected to decrease further to 15,922 by 2050, with an EAPC of −0.09% between 2021 and 2050. The ASDR was 518/100,000 in 1990 and 424/100,000 in 2021, with an EAPC of −0.68% from 1990 to 2021. It is expected to remain relatively stable at 419/100,000 by 2050, with an EAPC of −0.02% between 2021 and 2050. In 2021, the highest ASPRs were recorded in Senegal (34,421/100,000), Mali (34,233/100,000), and Pakistan (33,942/100,000). By 2050, Mali (35,070/100,000), Senegal (34,132/100,000), and Zambia (33,149/100,000) are projected to lead. For ASDR, Yemen (1405/100,000), Mozambique (1149/100,000), and Mali (1093/100,000) had the highest rates in 2021. By 2050, Yemen (1388/100,000), Mali (1181/100,000), and Mozambique (1177/100,000) are expected to remain the highest. SHAP values demonstrated that gender was the leading predictor of ID, with age and year showing negative contributions. Females aged 10 to 60 consistently showed higher prevalence and DALYs rates compared to males, with the under-5 age group having the highest rates for both. Additionally, men aged 80 and above exhibited a rapid increase in prevalence. Furthermore, the ASPR and ASDR were significantly higher in regions with a lower SDI, highlighting the greater burden of ID in less developed regions. Conclusions: ID remains a significant global health concern, with its burden projected to persist through 2050, particularly in lower-SDI regions. Despite declines in ASPR and ASDR, total cases and DALYs are expected to rise. SHAP analysis revealed that gender had the greatest influence on the model’s predictions, while both age and year showed overall negative contributions to ID risk. Children under 5, women under 60, and elderly men aged 80+ were the most vulnerable groups. These findings underscore the need for targeted interventions, such as improved nutrition, early screening, and addressing socioeconomic drivers through iron supplementation programs in low-SDI regions.

## 1. Introduction

Iron is an essential trace element for the human body and is involved in various physiological functions, including the synthesis of hemoglobin, oxygen transport, DNA synthesis, and muscle metabolism [1]. ID is widely recognized as one of the most prevalent forms of malnutrition. It can lead to anemia, which in turn affects the normal functioning of multiple bodily systems, impacting overall health and productivity [2]. Anemia is the most common nutritional deficiency worldwide, affecting 33% of non-pregnant women, 40% of pregnant women, and 42% of children globally [3,4,5]. Detecting ID during early pregnancy and infancy is crucial. ID in children under two years old can cause significant and irreversible impacts on brain development, which can negatively affect learning and academic performance. In pregnant women, ID can lead to anemia, reduced birth weight, and shortened pregnancy duration [6]. ID primarily occurs during rapid growth and developmental stages, such as early childhood, adolescence, and pregnancy, but it can also occur at other stages of life. For adults, ID can have negative effects, including fatigue, impaired physical performance, reduced work efficiency, and impacts on social activities [7].

In 2017, ID was the fifth most significant contributor to DALYs among women of reproductive age and the primary cause of DALYs in children aged 5–14 years of both genders combined in 2017 [8]. By 2019, ID was the seventh cause of burden among children aged <10 years and the eighth leading cause of global DALYs among individuals aged 10–24 years. A study conducted in 2019 reported that the prevalence cases and DALYs of children under 15 years were 391.49 million and 13.62 million, respectively, with an ASPR of 20.15 thousand/100,000 and an ASDR of 698.90/100,000 [9].

ID continues to pose a significant global health challenge, affecting vulnerable groups such as children and women. Accurately predicting the future burden of ID is crucial for guiding effective public health interventions and resource allocation. In the field of disease burden prediction, traditional models such as APC models and their Bayesian variations have long been used to assess the impact of age, time period, and cohort effects on disease incidence and mortality. However, these models struggle with capturing non-linear, multifactorial interactions and often oversimplify the complexity inherent in health data [10]. Furthermore, APC models can yield biased estimates, especially when trends in disease burden reverse or vary non-linearly, which poses significant challenges in providing accurate long-term forecasts [11]. To address these limitations, machine learning models, particularly XGBoost, have gained prominence. XGBoost has shown superior performance in disease prediction tasks, especially when compared to traditional models such as ARIMA and APC. For instance, a study comparing XGBoost with ARIMA in predicting human brucellosis demonstrated that XGBoost significantly outperformed ARIMA in terms of model accuracy and error reduction [12]. Another study on COVID-19 incidence prediction further supported this, showing that XGBoost was more accurate in capturing complex patterns and reducing forecast errors compared to ARIMA [13]. These results highlight XGBoost’s ability to model non-linear relationships and handle large datasets more effectively than traditional methods.

One of the challenges of using machine learning models like XGBoost is their “black-box” nature, where the internal workings and the contributions of individual variables are not easily interpretable. To address this, we incorporated SHAP, a tool grounded in cooperative game theory. SHAP provides interpretable insights by calculating how much each feature contributes to the model’s prediction, thus offering a transparent explanation for model decisions. This approach has been widely recognized for enhancing the explainability of machine learning models by using the concept of Shapley values from game theory to fairly distribute contributions among all features [14]. In recent years, the combination of XGBoost with SHAP has proven highly effective in disease prediction tasks. For example, SHAP has been used to predict 3-year all-cause mortality in patients with heart failure caused by coronary heart disease, identifying important factors such as age, natriuretic peptides, and drug use [15]. Additionally, SHAP has helped explain predictions in heart failure patients in intensive care units, improving model transparency and identifying key risk factors such as blood urea nitrogen levels [16]. By applying SHAP to our XGBoost model, we were able to quantify the contributions of key factors and provide actionable insights for addressing iron deficiency on a global scale. This advanced framework offers a robust and interpretable approach to predicting ID outcomes globally, providing valuable insights for public health policies.

The GBD 2021 provides a comprehensive framework for analyzing the global burden of diseases, injuries, and risk factors across various regions and populations. Utilizing a wide range of data sources, including health surveys, administrative reports, vital registries, and epidemiological studies, GBD 2021 estimates key health metrics such as prevalence, incidence, mortality, and DALYs [17,18,19,20]. It is an invaluable tool for understanding health trends and informing policy decisions aimed at improving population health. The GBD framework is particularly useful for studying nutritional deficiencies, including ID, which continues to pose significant challenges worldwide.

In this study, we used the GBD 2021 dataset to access the global, regional, and national prevalence and DALYs of ID from 1990 to 2021 and predicted the trends between 2022 and 2050. This analysis aimed to offer essential insights into the burden of ID and its projected future trends, enabling targeted interventions to reduce its impact on public health and address the challenges posed by ID.

## 2. Materials and Methods

### 2.1. Data Sources, Definitions

The data for our study, which analyzes the global burden of ID using prevalence and DALYs, were sourced from the GBD 2021 Results Tool (https://vizhub.healthdata.org/gbd-results/, accessed on 1 August 2024). This tool, developed by GBD collaborators, provides detailed analysis for hundreds of causes of death, diseases, injuries, and risk factors. Data on ID were extracted for the years 1990 to 2021, covering 204 countries and territories and 21 GBD regions. The data were stratified by year, age, and sex, including specific age groups: <1 year, 12–23 months, 2–4 years, 5–9 years, 10–14 years, 15–19 years, continuing in 5-year intervals up to 85+ years.

Population estimates from 1990 to 2021 were calculated by dividing the number of prevalent cases by the prevalence rate. Population data for 2022–2050 were obtained from the Institute for Health Metrics and Evaluation (IHME) [21]. The SDI, which combines measures of per-capita income, education levels, and fertility rates, was retrieved from the IHME for the years 1990 to 2021 [22]. Based on SDI quintiles, countries and territories were divided into five categories: low SDI, low–middle SDI, middle SDI, middle–high SDI, and high SDI. These quintiles allow for comparative analysis of disease burden across regions with varying socioeconomic development levels, helping to contextualize disparities in health outcomes related to ID.

### 2.2. Data Analysis

EAPCs were calculated to assess trends in ASPRs and DALYs for ID over time. EAPCs were derived from a linear regression model (Supplementary Materials File S1), where the natural logarithm of ASPRs or DALYs was modeled against time (year). An increasing trend was identified when the EAPC was greater than zero with a *p*-value of less than 0.05. Conversely, a decreasing trend was indicated when the EAPC was less than zero with a *p*-value of less than 0.05. If neither condition was met, the trend was considered stable. In addition, the SDI was introduced to analyze the relationship between SDI levels and both the prevalence and DALYs of ID. The correlation between SDI and the disease burden was explored to understand how socioeconomic factors influence the global and regional trends in ID burden.

A weighted correlation analysis and a weighted loess regression were conducted at the national level, with the ID prevalent cases used as weights for both analyses. This approach was designed to account for the varying burden of ID across countries, allowing for a more accurate assessment of the relationship between ID ASPR and anemia ASPR.

Using the GBD 2021 dataset for ID from 1990 to 2021, an XGBoost model was developed. The model used sex, age, year, and the natural logarithm of the population size as input features, with the log-transformed prevalence or DALYs rate (log(Prevalence or DALYs rate+1)) as the output. This model was constructed to predict the prevalence and DALYs rates for ID. The model formula and the features used are outlined as follows:

$${log(Prevalence\;or\;DALYs\;rate}_{y,c,s,a}+1)\;\sim s+a+y+{(\log\left(pnum\right))}_{y,c,s,a}$$

where:

y represents the calendar year; c denotes the country or region; s refers to sex (0 for female and 1 for male); a is the midpoint of each age group (e.g., a = 0.5 for ages < 1 year, 1.5 for ages 12–23 months, 3 for ages 2–4 years, continuing similarly for the other age brackets).

${Prevalence\;or\;DALYs\;rate}_{y,c,s,a}$ refers to the prevalence or DALYs rate in year y for nation/region c, sex s, and age group a. The ${log(Prevalence\;or\;DALYs\;rate}_{y,c,s,a}+1)$ transformation is applied to manage data skewness and handle cases where the rate is zero. The addition of 1 ensures that the logarithmic function is defined even when the prevalence or DALYs rate is zero.

${(\log\left(pnum\right))}_{y,c,s,a}$ represents the natural logarithm of population size in year y for a specific nation or region c, sex s, and age group a.

XGBoost, the machine learning model we used, constructs decision trees to make predictions. It works by adding new trees to the model iteratively, minimizing prediction errors in each step. The model’s objective function is made up of two parts: a loss function l, which measures how well the model predicts, and a regularization term Ω, which penalizes model complexity to prevent overfitting [23]. The corresponding formula is as follows:

$$L\left(\phi \right)=\sum_{i=1}^{n}l\left({\hat{y}}_{i},y_{i}\right)+\sum_{k=1}^{K}\Omega \left(f_{k}\right)$$

$$\Omega \left(f\right)=\gamma T+\frac{1}{2}\lambda \sum_{j=1}^{T}w_{j}^{2}$$

$L\left(\phi \right)$ is the overall objective function, composed of the sum of the loss function and a regularization term.

$l({\hat{y}}_{i},\;y_{i})$ is the loss function for each sample i, which measures the difference between the predicted value ${\hat{y}}_{i}$ and the actual value $y_{i}$.

$\Omega (f_{k})$ is the regularization term for tree k, which controls the complexity of the model to prevent overfitting.

T represents the total number of leaf nodes in the tree. More leaf nodes generally mean more complex trees.

$w_{j}$ is the weight of the j-th leaf node in the tree. Each leaf in the tree has a weight that contributes to the prediction.

γ is a regularization parameter that penalizes the number of leaf nodes in a tree. Larger γ values discourage trees from growing too large by increasing the cost associated with adding more leaf nodes.

λ is another regularization parameter that controls the magnitude of the leaf node weights $w_{j}$. Larger values of λ make the model more conservative by penalizing large leaf weights.

In building the XGBoost model, the data were first randomly split into a 70% training set and a 30% test set. On the training set, 5-fold cross-validation and grid search were applied to tune the hyperparameters, with root mean squared error (RMSE) used to evaluate various combinations of hyperparameters. Based on the optimal hyperparameter combination, the XGBoost model was trained, and its performance was evaluated on the test set by calculating the RMSE and Pearson’s correlation coefficient between the observed and predicted values.

The trained model was used to predict the prevalence and DALYs rates for the period 2022–2050. Based on population data and the standard population structure, the corresponding age-standardized rate (ASR) was also calculated. To estimate the 95% uncertainty intervals (UIs) for the predictions, 500 bootstrap samples were generated, with models trained on each sample. The 95% UI was defined using the 2.5th and 97.5th percentiles of the predicted outcomes.

To prevent overfitting during the training process for each bootstrap sample, early stopping was implemented. Early stopping works by monitoring the model’s performance on a validation set (in our case, out-of-bag (OOB) data) and halting training if there is no improvement in performance for a specified number of iterations. The early stop was set at 10, meaning that if the model’s validation performance did not improve for 10 consecutive boosting rounds, the training process was terminated to avoid overfitting and reduce unnecessary computation.

SHAP values helped us understand how different factors (like age, sex, and population size) influenced the model’s predictions for iron deficiency. It showed how much each factor increased or decreased the predicted risk, giving us a clear idea of which factors were the most important in the predictions [24]. The SHAP value for a feature j is computed as follows:

$$\phi_{j}=\sum_{S\subseteq F\backslash \{j\}}\frac{\left|S\right|!\left(\left|F\right|-\left|S\right|-1\right)!}{|F|!}[f\left(S\cup \left\{j\right\}\right)-f(S)]$$

$\phi_{j}$ is the SHAP value for feature j, indicating its contribution to the model’s predictions by considering its marginal impact across all possible subsets.

S⊆F\{j} represents all subsets of the full feature set F, excluding feature j.

$\frac{\left|S\right|!\left(\left|F\right|-\left|S\right|-1\right)!}{|F|!}$ is a weight factor based on the size of the subset S.

$\left|S\right|!$ denotes the factorial of the size of subset S, representing the number of possible orderings of features within the subset S.

(|F|−|S|−1)! accounts for the factorial of the features not included in subset S, adjusted for the exclusion of feature j.

$\left|F\right|!$ is the factorial of the total number of features, ensuring fair weight distribution across subsets of different sizes.

$f\left(S\cup \left\{j\right\}\right)$ is the prediction made by the model when feature j is added to subset S.

f(S) is the prediction based solely on the features in subset S, without feature j.

Using SHAP values allows for the interpretation of the significance of each feature in predicting the ID prevalence or DALYs rates, providing a clear view of how individual variables contribute to the overall model predictions.

An APC model was employed to decompose the effects on the prevalence and DALYs rates of ID into three distinct components: age, period, and birth cohort. This method approach enabled an evaluation of how each factor independently contributed to variations in prevalence and DALYs over time. To examine the period and birth cohort effects, rate ratios (RRs) were calculated, comparing the ID rates across different periods and birth cohorts relative to a designated reference group (Supplementary Materials File S2).

The APC analysis was conducted using the freely available APC Web Tool [25], while other processes, including data cleaning, analysis, and visualization, were performed using R (version 4.4.2). The ‘xgboost’ package was used for implementing the XGBoost model [26], and the ‘SHAPforxgboost’ package was applied for SHAP analysis [27].

## 3. Results

### 3.1. Sensitivity Analysis, Modeling Fitting, and Validation

To identify the optimal hyperparameters and assess the model’s predictive performance, the data were split into a 70% training set and a 30% test set. A grid search was conducted on the training set, focusing on three key hyperparameters: *max_depth* (maximum tree depth), *eta* (learning rate), and *nrounds* (number of boosting rounds). *max_depth* controls the complexity of the trees; while deeper trees can capture more intricate patterns, they also increase the risk of overfitting. *eta* determines the learning rate, influencing the convergence speed and stability of the model. *nrounds* defines the number of boosting rounds, affecting both the learning potential and the likelihood of overfitting (Supplementary Materials File S3) [23]. A 5-fold cross-validation was performed, using RMSE as the evaluation metric to explore various hyperparameter combinations (Supplementary Materials File S3). The configuration with the lowest RMSE was selected as optimal. This optimal configuration was then applied to the 30% test set to evaluate the model’s performance. For the ID prevalence and DALYs rates, the RMSE was 543.12/100,000 and 18.39/100,00, respectively. Furthermore, Pearson correlation coefficients between the observed and predicted values for the prevalence and DALYs rates were 1.00 (*p* < 0.001) and 1.00 (*p* < 0.001), respectively, highlighting the excellent predictive accuracy of the model (Figure S1).

### 3.2. Overall Burden of ID

#### 3.2.1. Prevalence of ID

**Figure 1:**
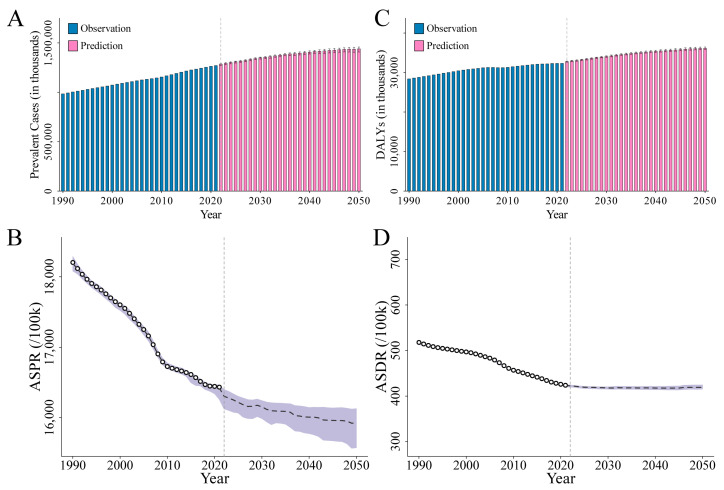
Number and ASR of ID from 1990 to 2050 at the global level. (**A**) Prevalent cases; (**B**) ASPR; (**C**) DALYs; (**D**) ASDR. Abbreviations: ASR, age-standardized rate; ID, iron deficiency; ASPR, age-standardized prevalence rate; DALYs, disability-adjusted life years; ASDR, age-standardized DALYs rate.

The global prevalent cases of ID in 1990 were 984.61 million, and it is projected to increase from 1270.64 million in 2021 to 1439.99 million in 2050 (Table 1, Figure 1A).

**Figure 2:**
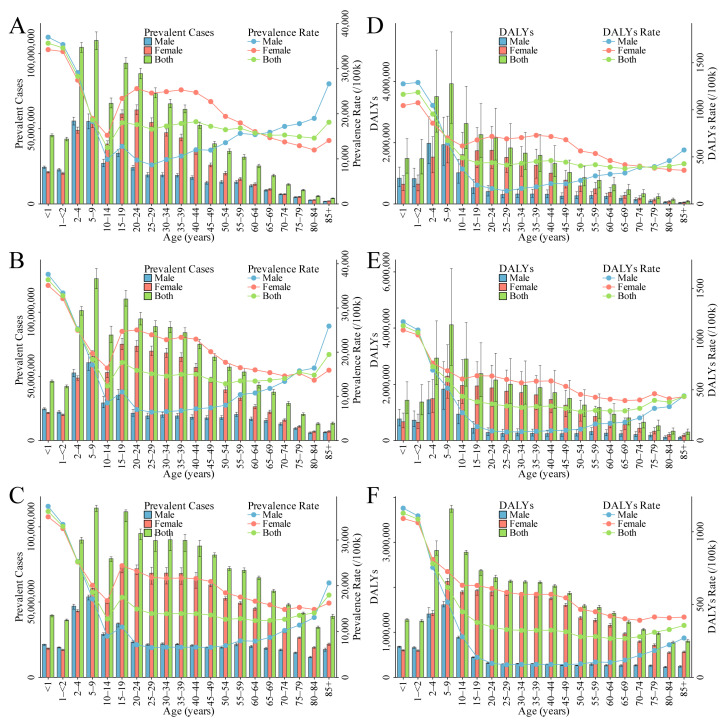
Number and rate of ID in 1990, 2021, and 2050 at the global level by gender and age groups. (**A**–**C**) Prevalent case and prevalence rate in 1990 (**A**), 2021 (**B**), and 2050 (**C**). (**D**–**F**) DALYs and DALYs rate in 1990 (**D**), 2021 (**E**), and 2050 (**F**). Abbreviations: ID, iron deficiency; DALYs, disability-adjusted life years.

The crude prevalence rates for 1990 and 2021 were 18,460/100,000 and 16,101/100,000, respectively, with a projected rate of 15,076/100,000 in 2050 (Table S1). Additionally, the ASPR decreased from 18,204/100,000 in 1990 to 16,433/100,000 in 2021, with a projection of 15,922/100,000 for 2050 (Table 1 and Table S2, Figure 1B). The EAPC in ASPR was −0.36 (95% CI: −0.38 to −0.34) from 1990 to 2021 and is predicted to be −0.09 (95% CI: −0.10 to −0.08) from 2021 to 2050 (Table 1 and Table S3). In 1990, 2021, and 2050, the highest prevalence rates were all observed in the <1 year and 12–23 months age groups, with rates of 35,652/100,000 and 34,601/100,000 in 1990, 36,402/100,000 and 32,780/100,000 in 2021, and projected rates of 36,253/100,000 and 32,968/100,000 in 2050, respectively. Furthermore, it was found that, within the age range of 10 to 60 years, the prevalence rate among females consistently remained higher than that of males, and after the age of 80, the prevalence rate in males increased rapidly. In the 80–84 years and 85+ years age groups, the prevalence rates were 14,602/100,000 and 18,125/100,000 in 1990, 14,801/100,000 and 19,469/100,000 in 2021, and are projected to be 14,061/100,000 and 17,964/100,000 by 2050, respectively (Table S4, Figure 2A−C, Figure S2 and S3A−C).

At the regional level, in 1990, the highest ASPRs were observed in South Asia, Central Sub-Saharan Africa, and Western Sub-Saharan Africa, with 36,430/100,000, 25,700/100,000, and 24,938/100,000, respectively. In 2021, the highest ASPRs were in South Asia, Western Sub-Saharan Africa, and Oceania, at 31,696/100,000, 26,159/100,000, and 21,731/100,000, respectively. By 2050, the highest ASPRs are projected to be in South Asia, Western Sub-Saharan Africa, and Oceania, with rates of 30,074/100,000, 26,045/100,000, and 21,120/100,000, respectively (Table 1 and Table S2). The highest EAPCs in ASPR were observed in High-income North America (EAPC: 0.25, 95% CI: 0.04 to 0.46), Western Sub-Saharan Africa (EAPC: 0.19, 95% CI: 0.15 to 0.23), and the Caribbean (EAPC: 0.03, 95% CI: −0.07 to 0.14) from 1990 to 2021. Between 2021 and 2050, the largest EAPCs in ASPR are predicted in East Asia (EAPC: 0.35, 95% CI: 0.32 to 0.38), High-income Asia Pacific (EAPC: 0.16, 95% CI: 0.12 to 0.20), and Western Sub-Saharan Africa (EAPC: −0.01, 95% CI: −0.01 to 0.01) (Table 1 and Table S3).

**Figure 3:**
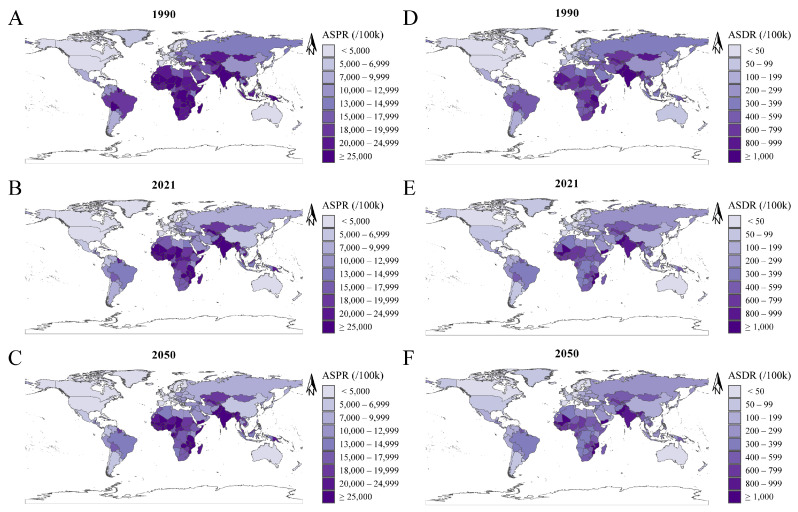
ASR of ID at the national level in 1990, 2021, and 2050. (**A**–**C**) ASPR in 1990 (**A**), 2021 (**B**), and 2050 (**C**). (**D**–**F**) ASDR in 1990 (**D**), 2021 (**E**), and 2050 (**F**). Abbreviations: ASR, age-standardized rate; ID, iron deficiency; ASPR, age-standardized prevalence rate; ASDR, age-standardized DALYs rate.

**Figure 4:**
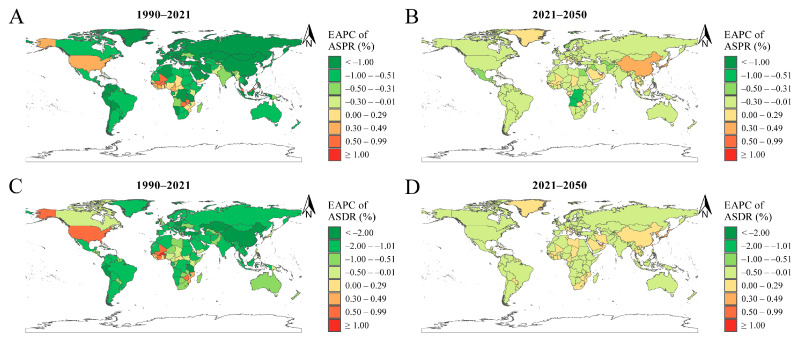
EAPC in ASR of ID at the national level from 1990 to 2021 and from 2021 to 2050. (**A**,**B**) EAPC in ASPR from 1990 to 2021 (**A**) and from 2021 to 2050 (**B**). (**C**,**D**) EAPC in ASDR from 1990 to 2021 (**C**) and from 2021 to 2050 (**D**). Abbreviations: EAPC, estimated annual percentage change; ASR, age-standardized rate; ID, iron deficiency; ASPR, age-standardized prevalence rate; ASDR, age-standardized DALYs rate.

At the national level, in 1990, the highest ASPRs were recorded in India, Nepal, and Pakistan, with rates of 36,853/100,000, 36,048/100,000, and 35,972/100,000, respectively. In 2021, the top ASPRs were found in Senegal (34,421/100,000), Mali (34,233/100,000), and Pakistan (33,942/100,000). By 2050, Mali (35,070/100,000), Senegal (34,132/100,000), and Zambia (33,149/100,000) are expected to have the highest ASPRs (Table 2 and Table S2, Figure 3A–C, and Video S1). Burkina Faso (EAPC: 1.00, 96% CI: 0.89 to 1.10), Zambia (EAPC: 0.92, 95% CI: 0.75 to 1.08), and Togo (EAPC: 0.80, 95% CI: 0.67 to 0.93) experienced the highest EAPCs in ASPR from 1990 to 2021. Meanwhile, projections indicate that the United Arab Emirates (EAPC: 0.70, 95% CI: 0.66 to 0.73), Qatar (EAPC: 0.55, 95% CI: 0.49 to 0.61), and China (EAPC: 0.36, 95% CI: 0.33 to 0.39) will exhibit the largest EAPCs in ASPR between 2021 and 2050 (Table S3, Figure 4A,B).

#### 3.2.2. DALYs of ID

Globally, in 1990, DALYs were recorded at 28.41 million. By 2021, this number had increased to 32.32 million, and it is projected to reach 36.15 million by 2050 (Table 1 and Figure 1C). Additionally, the ASDR decreased from 518/100,000 in 1990 to 424/100,000 in 2021, with a projection of 419/100,000 for 2050. The EAPC in ASDR was −0.68 (95% CI: −0.72 to −0.64) from 1990 to 2021 and is predicted to be −0.02 (95% CI: −0.04 to −0.01) from 2021 to 2050 (Table 1, Figure 1D, Tables S2 and S3). The highest DALYs rates were all observed in the <1 year, 12–23 months, and 2–4 years age groups in 1990, 2021, and 2050, with rates of 1164/100,000, 1186/100,000, and 955/100,000 in 1990, 1131/100,000, 1066/100,000, and 728/100,000 in 2021, and projected rates of 1128/100,000, 1087/100,000, and 781/100,000 in 2050, respectively. Furthermore, it was observed that within the age range of 10 to 60 years, the DALYs rate among women consistently remained higher than that among men (Table S4, Figure 2D–F, Figures S2 and S3D–F).

Regionally, High-income North America (EAPC: 0.70), Western Sub-Saharan Africa (EAPC: −0.08), and Oceania (EAPC: −0.08) had the highest EAPCs in ASDR from 1990 to 2021. Meanwhile, East Asia (EAPC: 0.21), High-income Asia Pacific (EAPC: 0.16), and the Caribbean (EAPC: 0.04) are projected to have the largest EAPC in ASDR from 2021 to 2050 (Table 1 and Table S3).

At the national level, in 1990, the countries with the highest ASDRs were Yemen (1440/100,000), Pakistan (1313/100,000), and Senegal (1269/100,000). By 2021, Yemen (1405/100,000), Mozambique (1149/100,000), and Mali (1093/100,000) emerged as the countries with the highest ASDRs. Projections for 2050 indicate that Yemen (1388/100,000), Mali (1181/100,000), and Mozambique (1177/100,000) will have the highest ASDRs (Table 2 and Table S2, Figure 3D–F, and Video S2). Between 1990 and 2021, Burkina Faso (EAPC: 1.45), Togo (EAPC: 0.98), and Côte d’Ivoire (EAPC: 0.97) recorded the largest EAPCs in ASDR. For the period from 2021 to 2050, Qatar (EAPC: 0.59), the United Arab Emirates (EAPC: 0.57), and the Democratic People’s Republic of Korea (EAPC: 0.41) are projected to have the highest EAPCs in ASDR (Table S3 and Figure 4C,D).

### 3.3. APC Analysis of ID Prevalence and DALYs

The APC model was used to analyze the age, period, and birth cohort effects on ID prevalence and DALYs. The age effect analysis revealed that individuals under 5 years of age had the highest prevalence rates (31,919/100,000). Additionally, the data showed that females aged 10 to 84 years had a higher prevalence rate of ID compared to males. Regarding DALYs, the highest rates were also observed in children under five (1092/100,000), with females over the age of 10 exhibiting higher ID-related DALYs rates than their male counterparts (Table S5, Figure S4A,D).

The period effect results indicated that from 1990–1994 compared to 2045–2049, the prevalence period rate ratio (RR) for females remained relatively stable, while for males, it showed a decreasing trend. The period RR of prevalence for males decreased from 1.25 in 1990–1994 to 0.84 in 2045–2049. A similar trend was observed in the RR for DALYs, with the male DALYs period RR declining from 1.50 in 1990–1994 to 0.74 in 2045–2049 (Table S6, Figure S4B,E).

The birth cohort effect for female prevalence showed relatively minor variations from 1905 to 2045. In contrast, males exhibited an overall decreasing trend during this period, with the birth cohort RR for male prevalence declining from 1.61 in 1905 to 0.77 in 2045. Similarly, the birth cohort effect on DALYs mirrored the trend observed in prevalence, with the birth cohort RR for male DALYs decreasing from 2.72 in 1905 to 0.62 in 2045 (Table S7, Figure S4C,F).

### 3.4. SHAP Analysis on the Variables Influencing ID Prevalence and DALYs Rates

**Figure 5:**
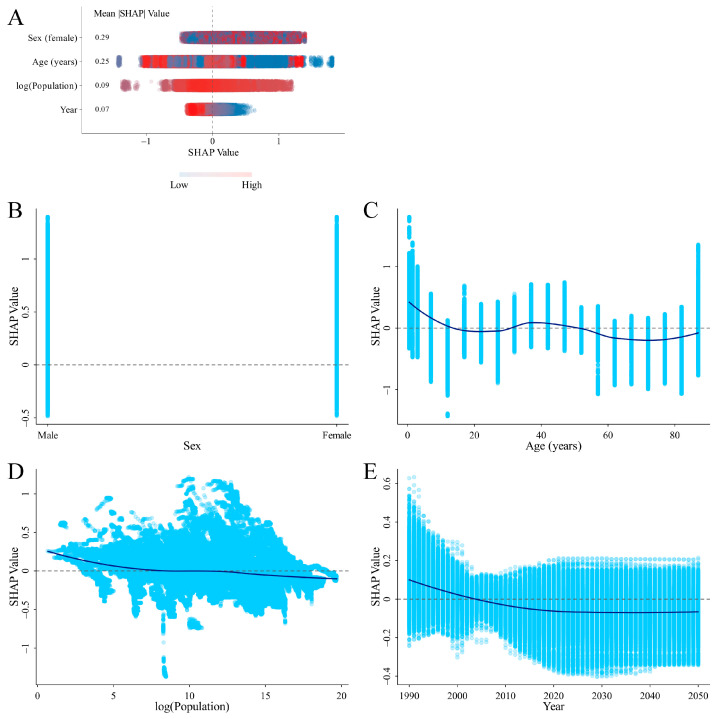
SHAP summary plot of feature contributions ranked by mean |SHAP| values and SHAP dependence plots for each feature in the XGBoost model predicting ID prevalence rate. (**A**) Summary plot. SHAP values measure the contribution of each feature to the prediction for a given data point. A positive SHAP value means the feature increases the predicted risk of ID, while a negative SHAP value means the feature decreases the predicted risk. The magnitude of the SHAP value indicates how strongly the feature affects the prediction. Larger absolute SHAP values (further from zero) mean the feature has a stronger influence, either increasing or decreasing the predicted outcome. The mean absolute SHAP values (|SHAP|) reflect the overall importance of each feature across the entire dataset. Features with higher mean |SHAP| values are more important to the model’s predictions. In this SHAP summary plot figure, the features are ordered from most important to least important based on their mean |SHAP| values. The color of the points represents the normalized value of each feature. Red indicates high feature values, while blue indicates low feature values. This color gradient helps explain how different values of the feature affect the prediction. For example, if the red points (high feature values) are mostly on the positive side of the SHAP axis, it means the high values of that feature increase the predicted risk of ID. Conversely, if the blue points (low feature values) cluster on the negative side, it suggests that the low values of that feature decrease the predicted risk. (**B**–**E**) The dependence plot showing the contribution of gender (**B**), age (**C**), year (**D**), and log (population) (**E**). Abbreviations: SHAP, SHapley Additive exPlanations; ID, iron deficiency.

**Figure 6:**
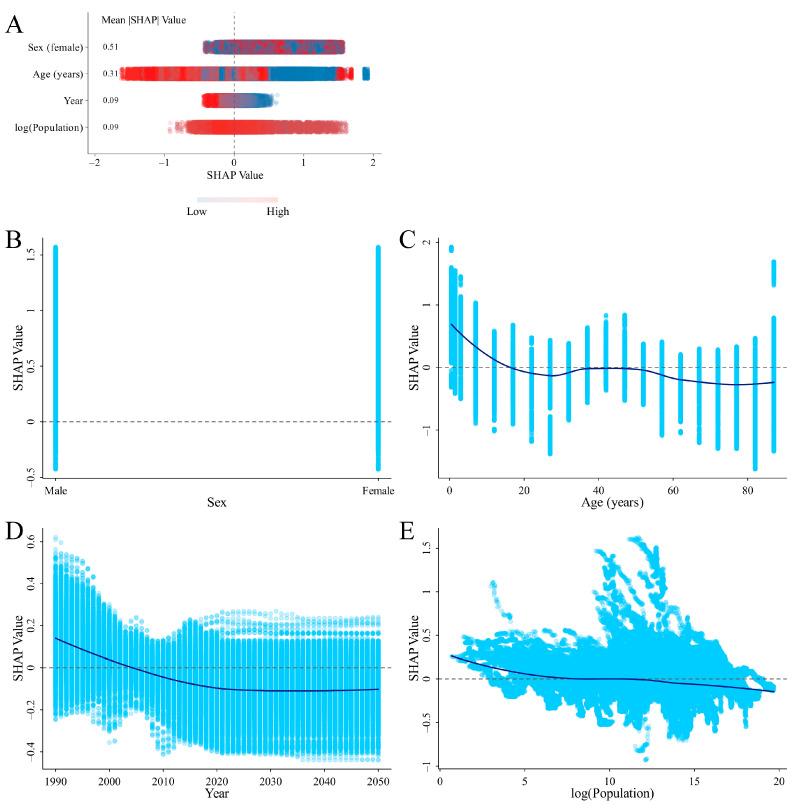
SHAP summary plot of feature contributions ranked by mean |SHAP| values and SHAP dependence plots for each feature in the XGBoost model predicting ID DALYs rate. (**A**) Summary plot. The interpretation of SHAP values, including the significance of positive and negative SHAP values, the magnitude of SHAP values, the ranking by mean absolute SHAP values (|SHAP|), and the color gradient (red for high values, blue for low values), follows the same explanation provided in Figure 5. (**B**–**E**) The dependence plot showing the contribution of gender (**B**), age (**C**), year (**D**), and log (population) (**E**). Abbreviations: SHAP, SHapley Additive exPlanations; ID, iron deficiency; DALYs, disability-adjusted life years.

SHAP values show how much each feature influences individual predictions. Since features can have both positive and negative effects, we calculated the average of their absolute SHAP values to see how important each feature is overall. Two SHAP summary plots were generated, highlighting four key features ranked by their importance in predicting the ID prevalence and DALYs rates. The analysis indicated that gender was the most influential factor, while age, log (population), and year generally had a negative effect on the predictions. The dependence plot also showed that individuals under 10 years old and periods before 2005 were associated with a higher risk of ID (Figure 5 and Figure 6).

Nationally, the contributing factors were analyzed based on SHAP values for the top three countries with the largest ASPR (Senegal, Mali, and Pakistan) or ASDR (Yemen, Mozambique, and Mali) in 2021. The analysis revealed that gender was the most significant contributing factor in the predictive model, while age generally showed a negative association with ID prevalence in these countries (Figures S5–S10).

### 3.5. Correlation between SDI and ASPR or ASDR of ID

**Figure 7:**
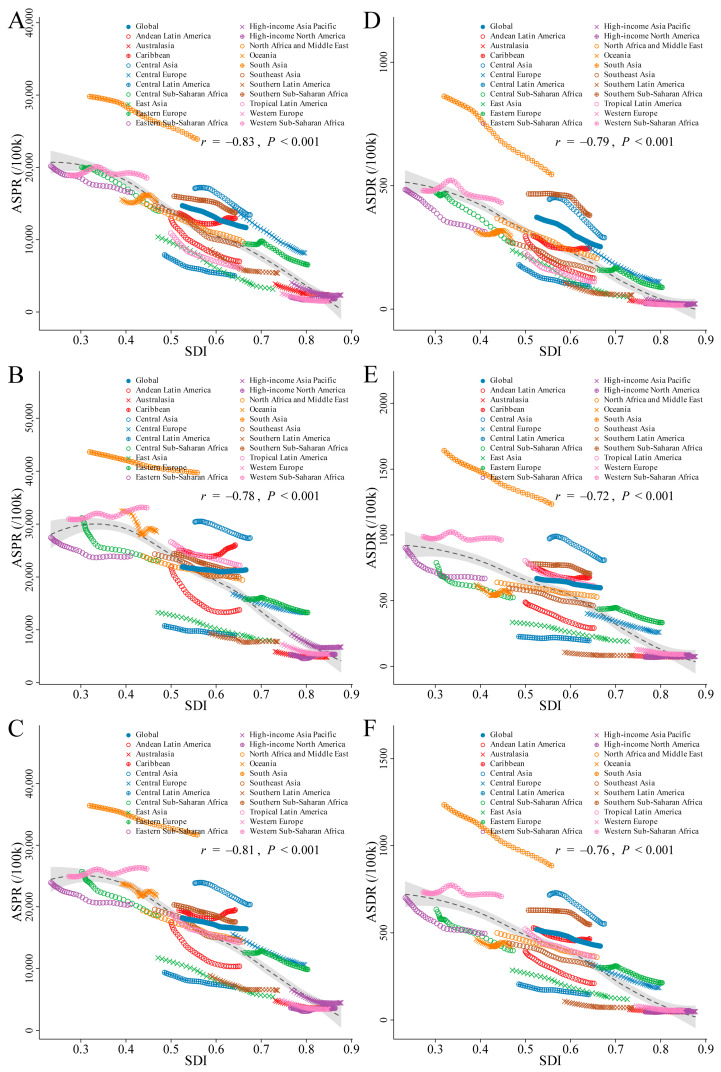
Correlation analysis between the SDI and ID ASR at global and regional levels by gender. (**A**–**C**) ASPR for male (**A**), female (**B**), and both genders combined (**C**). (**D**–**F**) ASDR for male (**D**), female (**E**), and both genders combined (**F**). Abbreviations: SDI, Socio-Demographic Index; ID, iron deficiency; ASR, age-standardized rate; ASPR, age-standardized prevalence rate; ASDR, age-standardized DALYs rate.

The correlation between the SDI and both ASPR and ASDR at the regional level, stratified by gender, was analyzed over the period from 1990 to 2021. The analysis revealed a significant negative correlation between the ASPR and SDI, indicating a decline in the ASPR with increasing SDI (male: *r* = −0.83, *p* < 0.001; female: *r* = −0.78, *p* < 0.001; both genders combined: *r* = −0.81, *p* < 0.001). Similarly, the ASDR showed a general downward trend with rising SDI during the same period (males: *r* = −0.79, *p* < 0.001; females: *r* = −0.72, *p* < 0.001; both genders combined: *r* = −0.76, *p* < 0.001) (Figure 7).

### 3.6. Correlation between the ASPR of ID and Anemia

**Figure 8:**
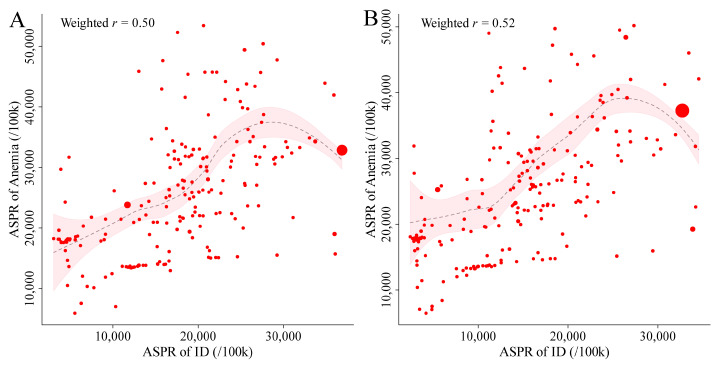
Weighted correction and weighted loess regression between the ASPR of ID and the ASPR of anemia in 1990 (**A**) and 2021 (**B**), with prevalent cases as weights. Point size represents the weighting. Abbreviations: ASPR, age-standardized prevalence rate; ID, iron deficiency.

The correlation between the ASPR of ID and anemia at the national level was examined for the years 1990 and 2021. Scatter plots were created, and the weighted correlation coefficients were calculated using the prevalent cases of ID as weights. Overall, an upward trend in anemia ASPR was observed with an increasing ASPR of ID. The weighted correlation coefficients for 1990 and 2021 were 0.50 and 0.52, respectively (Figure 8).

## 4. Discussion

In this study, the burden of ID was analyzed based on the GBD 2021 dataset, including prevalence and DALYs. The results indicated that from 1990 to 2021, both the ASPR and ASDR showed a decreasing trend. For the period from 2021 to 2050, the ASPR is projected to continue its decline, while the ASDR is expected to remain relatively stable. The APC model was used to analyze ID prevalence and DALYs. The effect of specific variables on ID prevalence and DALYs was demonstrated using SHAP values. Additionally, the correlation between the SDI and ID was analyzed, along with a preliminary exploration of the relationship between ID ASPR and anemia ASPR.

### 4.1. The Burden of ID: Groups with Higher Burden among Children, Women, and Elderly Men

Our results showed that children under 5 years old were particularly susceptible to ID, which was due to rapid growth, insufficient dietary iron, low birth iron stores, and breastfeeding practices. Infants and young children experienced rapid physical and neurological development, which increased their iron needs. If their diet did not supply adequate iron, they were at a higher risk of deficiency [28]. Many young children are transitioned to solid foods that may not be rich in iron or are given cow’s milk, which is low in iron and can interfere with iron absorption. This dietary insufficiency is a leading cause of ID in this age group [29]. Infants born prematurely or with low birth weight often have lower iron stores, putting them at a higher risk of ID early in life. This is especially true in regions with high rates of maternal ID [30]. Breast milk contains relatively low amounts of iron, and prolonged exclusive breastfeeding without iron supplementation or the introduction of iron-rich foods can lead to iron deficiency [31].

In the age range of 10 to 60 years, females were the primary affected group compared to males. Gender differences in ID were driven by menstruation, pregnancy, dietary habits, and higher iron requirements in females. Women of reproductive age are more prone to iron deficiency due to regular menstrual blood loss, which can deplete iron stores. This makes iron deficiency more common in females compared to males [32]. During pregnancy, women’s iron requirements increase significantly to support fetal development and increase blood volume. This often leads to ID, particularly in populations with insufficient iron intake [33]. In some cultures, men and women have different dietary patterns. Women, in certain cases, may consume fewer iron-rich foods or follow diets that inhibit iron absorption, increasing their risk of deficiency compared to men [34]. Women generally have higher iron requirements due to the factors mentioned above, but they may not always compensate for this through diet or supplements, making them more susceptible to iron deficiency anemia [35].

Men aged 80 and older are particularly vulnerable to ID. After the age of 80, iron deficiency becomes more common due to a variety of factors, including reduced dietary intake, decreased absorption of iron due to changes in gastrointestinal function, and the higher likelihood of chronic health conditions such as gastrointestinal bleeding or ulcers. Furthermore, the use of certain medications, including proton pump inhibitors and anticoagulants, can interfere with iron metabolism, compounding the risk in elderly men [36]. As a result, elderly men are at a greater risk of chronic ID, which can contribute to fatigue, cognitive decline, and the worsening of underlying health conditions. Unlike younger populations, ID in elderly men may not manifest through clear symptoms, such as fatigue, but can instead be linked to less obvious health issues, such as deteriorating cognitive function or worsening of pre-existing health conditions [37,38]. These subtler symptoms often delay diagnosis, further exacerbating the condition.

To mitigate the burden of ID in high-risk groups such as children, women, and elderly men, several targeted interventions are proposed. For children under 5 years old, particularly those born prematurely or with low birth weight, early iron supplementation is crucial to address the risk of iron depletion. Promoting the consumption of iron-fortified foods, such as cereals and formula, is particularly important in regions with high maternal ID rates. Caregivers should be educated on introducing iron-rich complementary foods, such as lean meats, legumes, and iron-fortified grains, during the weaning process to ensure adequate iron intake. Iron supplementation is also recommended for high-risk infants, particularly those transitioning from breastfeeding to complementary foods, as it helps prevent ID [39].

For women, particularly those of reproductive age and pregnant women, iron supplementation programs are recommended to counteract menstrual blood loss and increased iron demands during pregnancy. Routine prenatal iron supplements should be provided to support fetal development and increase maternal blood volume [40]. Additionally, community-based education programs that promote the consumption of iron-rich foods, paired with vitamin C to enhance absorption, should be implemented, and health programs should discourage the consumption of tea or coffee during meals, as they inhibit iron absorption [41].

For elderly men, regular screening for ID should be integrated into routine health checks, particularly for those with chronic illnesses or on medications that interfere with iron metabolism. Public health programs should also focus on educating elderly men about the importance of maintaining a balanced diet rich in iron and consider iron supplementation where necessary [36,42].

Finally, public health programs should consider fortifying staple foods (such as flour or rice) with iron to improve population-wide iron intake, particularly in areas with high rates of deficiency. Fortification has been proven to improve iron status significantly in at-risk populations [43]. Combining these strategies is essential for reducing the prevalence of iron deficiency among vulnerable groups.

### 4.2. The Impact of Social and Economic Development on ID Burden: ASPR and ASDR Trends across SDI Level

The inverse correlation between the SDI and both the ASPR and ASDR suggests that higher social and economic development, as indicated by a higher SDI, is associated with a lower disease ASPR and ASDR. This could be due to a range of factors, including improved healthcare access, better nutrition, and more advanced medical treatments in regions with a higher SDI [44]. Improved healthcare systems in higher SDI countries allow for preventive healthcare measures, such as early screenings for ID, and public health interventions like food fortification programs. However, an elevated ASPR and ASDR in certain high-SDI regions, such as Western Europe, may reflect the influence of non-communicable diseases (NCDs), aging populations, and lifestyle factors like processed food consumption, which are common in more developed countries. The growing prevalence of NCDs, such as diabetes and cardiovascular diseases, in high-SDI regions also contributes to persistent rates of ID [36,38,45,46]. Addressing the ID burden in high-SDI regions requires a multifaceted approach. Public health campaigns must promote nutritional education that encourages the consumption of iron-rich foods while reducing reliance on processed foods. This can be complemented by regular screenings for iron deficiency, particularly among high-risk groups such as the elderly and individuals with NCDs [47,48,49].

Regions with a lower SDI, such as Sub-Saharan Africa and South Asia, face disproportionately high rates of ID due to a combination of socioeconomic, dietary, and healthcare-related challenges [50,51]. These regions experience higher prevalence and death rates associated with ID, driven by limited healthcare resources, inadequate sanitation, widespread malnutrition, and a high burden of infectious diseases, which all exacerbate iron loss and affect iron absorption.

In Sub-Saharan Africa, several key factors drive the high burden of ID. Chronic hookworm infections are common in areas with poor sanitation, leading to gastrointestinal blood loss and exacerbating iron deficiency. The severity of ID is often proportional to the intensity of the worm burden [52]. Moreover, the region is heavily affected by malaria, which contributes to iron depletion through hemolysis and increased iron utilization. Malaria-induced anemia and ID are particularly prevalent among children and pregnant women, who are most vulnerable to these effects [53]. Another significant contributor to the ID burden in Sub-Saharan Africa is food insecurity and the reliance on monotonous diets, primarily consisting of staples like maize, cassava, and millet. These foods are low in bioavailable iron and high in phytates, which inhibit iron absorption, further increasing the risk of ID [54].

South Asia faces a high burden of ID due to similar overlapping factors. The region suffers from poor dietary diversity, with many populations relying on plant-based diets that are low in bioavailable iron and rich in phytates, which inhibit the absorption of non-heme iron. Gender disparities in South Asia further exacerbate this issue, as women of reproductive age are disproportionately affected due to higher iron requirements during menstruation, pregnancy, and childbirth. Cultural norms and limited access to healthcare also contribute to higher vulnerability to ID among women [55]. In addition to poor diets, widespread malnutrition and infectious diseases, such as tuberculosis and diarrheal diseases, are common in the region, leading to nutrient malabsorption and exacerbating ID [56,57,58].

Both Sub-Saharan Africa and South Asia also face significant socioeconomic challenges, including poverty, food insecurity, and limited healthcare infrastructure, which hinder efforts to reduce ID rates. Many populations in these regions lack access to iron-fortified foods due to high costs and distribution challenges, while healthcare systems often lack resources, limiting the effectiveness of interventions such as iron supplementation and deworming programs. Furthermore, the lack of sanitation infrastructure contributes to the ongoing transmission of infections like hookworm and malaria, which further worsen ID, while limited access to clean water exacerbates malnutrition and poor health overall [59,60,61].

Regions with a lower SDI, such as South Asia, struggle with higher prevalence and death rates due to limited healthcare resources, poor sanitation, malnutrition, and the burden of infectious diseases. In low- and lower-middle-income countries, chronic hookworm infection promotes gastrointestinal bleeding, and the severity of ID is proportional to the worm burden [62].

To effectively address rising ID rates in these regions, targeted interventions must be multifaceted and sustainable. Deworming programs are essential for reducing gastrointestinal blood loss caused by hookworm infections, particularly in children and pregnant women [63]. Iron supplementation should be expanded to vulnerable populations, but to ensure long-term success, these efforts must be complemented by broader strategies such as food fortification programs. For instance, fortifying staples like flour and salt with iron could significantly reduce ID rates, especially where dietary diversity is low [64]. In addition to nutritional interventions, improving healthcare and sanitation infrastructure is critical to addressing the root causes of malnutrition and reducing the spread of infectious diseases. Enhancing access to clean water and improving sanitation systems would not only prevent infections that exacerbate ID but also promote overall health [65]. Beyond healthcare, socioeconomic reforms play a pivotal role. Addressing poverty, improving education, and enhancing economic opportunities can significantly reduce the risk of malnutrition and ID in low-income regions [66]. Programs aimed at increasing income and food security are necessary to create a sustainable environment where nutritional interventions can succeed. Without addressing these structural barriers, nutritional interventions alone will have a limited impact. In conclusion, a comprehensive strategy that includes deworming, iron supplementation, food fortification, healthcare improvements, and socioeconomic reforms is essential to tackling rising ID rates, particularly in low- and middle-income countries. This multifaceted approach will ensure that interventions are effective in both the short and long term.

Projections emphasize the need for early and proactive interventions to mitigate rising ID. Between 1990 and 2021, the highest EAPC in ASPR was observed in regions like High-income North America and Western Sub-Saharan Africa, reflecting disparities in ID prevalence. From 2021 to 2050, East Asia and the High-income Asia Pacific regions are expected to have the largest EAPC in ASPR, driven by rapid urbanization and dietary shifts towards processed foods lacking essential micronutrients like iron [67,68,69].

### 4.3. SHAP Analysis for Identifying Critical Factors Influencing ID

In our study, SHAP analysis demonstrated the effectiveness of SHAP values in capturing the true impact of variables on ID. The dependence plots for SHAP values related to age and year exhibited patterns similar to those seen in the APC model, supporting the reliability of SHAP analysis in assessing variable influence. Gender was identified as the leading factor influencing ID risk, with women experiencing a markedly higher vulnerability. Age, in contrast, negatively contributed to ID risk, underscoring the heightened susceptibility of younger individuals, particularly children, to ID. These findings underscore the capacity of the SHAP analysis to precisely identify and quantify the factors driving ID. By applying SHAP analysis, the most influential risk factors can be better prioritized, enabling more effective public health strategies to reduce ID across different population groups.

### 4.4. Implications of the Correlation between ID and Anemia

The analysis of the correlation between the ASPR of ID and anemia in 1990 and 2021, with weighted correlation coefficients of 0.50 and 0.52, respectively, suggests a strong association between the two conditions. The slight increase in correlation over time indicates that ID is closely linked to anemia prevalence, though other factors, such as additional micronutrient deficiencies and infections, may also contribute to anemia outcomes.

While this correlation highlights the significant role of ID in the broader anemia burden, it underscores the need for multifaceted public health strategies. Addressing ID is important, while broader interventions that also target other nutritional and health factors are necessary to more effectively reduce anemia and its associated health consequences.

### 4.5. Challenges in Modeling ID with XGBoost and SHAP

Our model, utilizing the XGBoost algorithm alongside SHAP for feature interpretation, effectively predicted ID prevalence and DALYs based on key demographic variables such as gender, age, year, and population size. However, this approach has several limitations. The model is built on a limited set of demographic inputs without incorporating crucial factors like income, healthcare access, and nutrition, which are important determinants of ID variability. Additionally, it does not account for environmental and cultural factors, such as dietary habits and food availability, potentially reducing its accuracy in different regions. The model also lacks data on public health interventions and health conditions affecting iron metabolism, such as iron supplementation programs or infections, limiting its ability to capture temporal and health-related changes. Lastly, the model’s ability to generalize across diverse regions is constrained by the absence of region-specific factors that are critical for understanding local ID dynamics. Expanding the model to include broader socioeconomic, health, and environmental variables would improve both its predictive accuracy and applicability across different contexts.

## 5. Conclusions

ID remains a significant global health challenge, with its burden projected to persist and grow, particularly in low-SDI regions. Although the ASPR and ASDR are expected to decline slightly, total prevalence cases and DALYs will rise due to population growth and persistent inequalities. SHAP analysis identified gender and age as major predictors, with children under 5, women under 60, and men aged 80+ years being the most vulnerable. Lower SDI regions consistently show a higher ASPR and ASDR, underscoring the need for targeted interventions in these areas. To reduce the burden of ID, a comprehensive public health approach is needed, including iron supplementation programs, early screening, nutritional education, and food fortification, especially in low-SDI regions.

## Figures and Tables

**Table 1:** Prevalence and DALYs for ID in 1990, 2021, and 2050, and EAPC in ASRs from 1990 to 2021 and from 2021 to 2050. Abbreviations: DALYs, disability-adjusted life years; ID, iron deficiency; EAPC, estimated annual percentage change; ASRs, age-standardized rates; SDI, Socio-Demographic Index; CI: confidence interval.

Location	Number in Thousands (ASR per 100,000)			EAPC (95% CI) (%)	
	1990	2021	2050	1990–2021	2021–2050
Prevalence					
Overall	984,607.52 (18,204)	1,270,637.34 (16,433)	1,439,994.05 (15,922)	−0.36 (−0.38 to −0.34)	−0.09 (−0.10 to −0.08)
Sex					
Male	396,759.00 (14,700)	444,691.17 (11,675)	484,424.28 (11,042)	−0.78 (−0.82 to −0.75)	−0.18 (−0.19 to −0.16)
Female	587,848.51 (21,892)	825,946.17 (21,336)	955,569.76 (21,000)	−0.10 (−0.13 to −0.08)	−0.02 (−0.03 to −0.02)
Region					
Andean Latin America	7082.95 (17,489)	6821.55 (10,382)	8028.49 (9728)	−1.81 (−1.97 to −1.64)	−0.22 (−0.23 to −0.22)
Australasia	943.61 (4832)	1110.59 (3620)	1268.77 (3285)	−0.91 (−1.04 to −0.78)	−0.24 (−0.28 to −0.20)
Caribbean	7053.05 (19,457)	8961.35 (19,491)	8457.69 (19,163)	0.03 (−0.07 to 0.14)	−0.04 (−0.05 to −0.03)
Central Asia	17,249.54 (23,839)	19,799.63 (20,386)	23,470.23 (20,014)	−0.64 (−0.70 to −0.58)	−0.06 (−0.06 to −0.06)
Central Europe	18,944.21 (15,569)	11,687.33 (10,623)	8902.88 (10,318)	−1.28 (−1.35 to −1.21)	−0.10 (−0.11 to −0.09)
Central Latin America	16,343.81 (9372)	17,116.68 (7011)	20,406.94 (6691)	−0.87 (−0.94 to −0.81)	−0.14 (−0.16 to −0.12)
Central Sub-Saharan Africa	14,108.07 (25,700)	25,333.57 (18,599)	39,115.37 (16,030)	−1.05 (−1.11 to −1.00)	−0.49 (−0.51 to −0.46)
East Asia	139,772.15 (11,751)	83,926.60 (5418)	84,822.11 (5883)	−2.71 (−2.80 to −2.62)	0.35 (0.32 to 0.38)
Eastern Europe	28,550.56 (12,586)	20,900.01 (9869)	17,403.56 (9623)	−0.96 (−1.10 to −0.83)	−0.07 (−0.08 to −0.06)
Eastern Sub-Saharan Africa	48,124.79 (23,921)	89,598.69 (20,374)	158,241.73 (19,802)	−0.57 (−0.61 to −0.53)	−0.08 (−0.10 to −0.06)
High-income Asia Pacific	11,366.25 (6551)	9661.41 (4459)	8807.25 (4607)	−1.09 (−1.33 to −0.85)	0.16 (0.12 to 0.20)
High-income North America	10,707.62 (3676)	13,933.83 (3581)	14,606.84 (3420)	0.25 (0.04 to 0.46)	−0.13 (−0.16 to −0.10)
North Africa and Middle East	67,719.38 (19,409)	88,916.15 (14,363)	114,782.16 (13,217)	−0.91 (−0.94 to −0.88)	−0.24 (−0.26 to −0.21)
Oceania	1544.13 (23,765)	3038.61 (21,731)	4750.17 (21,120)	−0.23 (−0.31 to −0.15)	−0.11 (−0.11 to −0.10)
South Asia	393,349.18 (36,430)	569,464.64 (31,696)	615,459.06 (30,074)	−0.48 (−0.50 to −0.46)	−0.18 (−0.20 to −0.17)
Southeast Asia	88,446.72 (19,578)	99,767.83 (14,574)	111,458.90 (13,929)	−1.05 (−1.13 to −0.98)	−0.16 (−0.17 to −0.15)
Southern Latin America	4380.46 (8888)	4061.43 (6454)	4061.79 (5972)	−0.94 (−1.10 to −0.77)	−0.26 (−0.28 to −0.23)
Southern Sub-Saharan Africa	11,097.39 (20,322)	14,257.31 (17,563)	18,328.74 (17,133)	−0.50 (−0.52 to −0.49)	−0.06 (−0.07 to −0.05)
Tropical Latin America	29,083.48 (18,890)	31,992.38 (14,253)	31,131.27 (14,046)	−0.88 (−0.93 to −0.83)	−0.04 (−0.05 to −0.03)
Western Europe	17,794.45 (4857)	14,795.69 (3418)	14,135.56 (3340)	−1.05 (−1.21 to −0.89)	−0.08 (−0.10 to −0.05)
Western Sub-Saharan Africa	50,945.72 (24,938)	135,492.08 (26,159)	259,429.69 (26,045)	0.19 (0.15 to 0.23)	−0.01 (−0.01 to −0.01)
High SDI	53,685.35 (6152)	51,611.55 (4579)	59,591.16 (4510)	−0.78 (−0.94 to −0.61)	0.03 (−0.01 to 0.06)
High–middle SDI	128,441.90 (12,197)	102,978.16 (8005)	117,788.73 (7779)	−1.50 (−1.56 to −1.44)	−0.10 (−0.11 to −0.09)
Middle SDI	295,665.49 (17,387)	324,486.32 (13,564)	414,938.03 (12,840)	−0.83 (−0.85 to −0.81)	−0.19 (−0.20 to −0.17)
Low–middle SDI	349,563.99 (30,212)	485,130.96 (25,686)	716,530.29 (24,659)	−0.54 (−0.56 to −0.52)	−0.14 (−0.15 to −0.13)
Low SDI	156,394.36 (30,844)	305,505.41 (27,288)	617,457.33 (26,374)	−0.44 (−0.47 to −0.41)	−0.11 (−0.11 to −0.10)
DALYs					
Overall	28,406.13 (518)	32,315.75 (424)	36,131.94 (419)	−0.68 (−0.72 to −0.64)	−0.02 (−0.04 to −0.01)
Sex					
Male	10,389.00 (372)	9382.60 (253)	9515.37 (242)	−1.32 (−1.40 to −1.24)	−0.15 (−0.17 to −0.12)
Female	18,017.13 (668)	22,933.15 (598)	26,616.56 (603)	−0.37 (−0.39 to −0.34)	0.05 (0.04 to 0.06)
Region					
Andean Latin America	167.41 (394)	135.59 (209)	160.23 (199)	−2.18 (−2.23 to −2.13)	−0.14 (−0.16 to −0.12)
Australasia	11.93 (59)	14.56 (46)	16.48 (43)	−0.78 (−0.91 to −0.66)	−0.22 (−0.25 to −0.19)
Caribbean	193.90 (527)	209.49 (465)	192.52 (469)	−0.44 (−0.54 to −0.34)	0.04 (0.03 to 0.05)
Central Asia	529.88 (716)	538.54 (551)	626.23 (544)	−1.07 (−1.18 to −0.96)	−0.05 (−0.05 to −0.05)
Central Europe	396.51 (329)	203.38 (184)	154.75 (177)	−1.98 (−2.07 to −1.90)	−0.17 (−0.19 to −0.14)
Central Latin America	369.79 (205)	352.33 (145)	403.49 (138)	−0.99 (−1.06 to −0.92)	−0.19 (−0.21 to −0.17)
Central Sub-Saharan Africa	378.66 (633)	571.64 (398)	948.47 (386)	−1.45 (−1.56 to −1.33)	−0.11 (−0.12 to −0.10)
East Asia	3300.13 (285)	1772.46 (117)	1650.65 (123)	−3.12 (−3.23 to −3.01)	0.21 (0.19 to 0.23)
Eastern Europe	666.54 (299)	431.67 (212)	346.42 (205)	−1.42 (−1.60 to −1.23)	−0.14 (−0.15 to −0.13)
Eastern Sub-Saharan Africa	1523.52 (700)	2288.52 (496)	3827.83 (479)	−1.22 (−1.30 to −1.14)	−0.11 (−0.12 to −0.10)
High-income Asia Pacific	111.77 (66)	110.03 (47)	102.00 (50)	−0.87 (−1.07 to −0.66)	0.16 (0.12 to 0.21)
High-income North America	137.08 (47)	200.97 (53)	214.45 (51)	0.70 (0.55 to 0.85)	−0.10 (−0.11 to −0.09)
North Africa and Middle East	1841.63 (499)	2241.35 (360)	2933.00 (351)	−1.03 (−1.05 to −1.01)	−0.07 (−0.08 to −0.06)
Oceania	31.50 (465)	60.72 (419)	92.57 (407)	−0.08 (−0.18 to 0.02)	−0.11 (−0.11 to −0.10)
South Asia	13,548.86 (1235)	15,671.28 (885)	16,495.17 (837)	−1.09 (−1.13 to −1.04)	−0.20 (−0.22 to −0.18)
Southeast Asia	2072.55 (440)	2096.97 (310)	2300.82 (309)	−1.16 (−1.20 to −1.13)	−0.06 (−0.08 to −0.05)
Southern Latin America	51.84 (106)	43.95 (69)	42.74 (62)	−1.21 (−1.41 to −1.00)	−0.32 (−0.34 to −0.29)
Southern Sub-Saharan Africa	362.16 (629)	445.09 (546)	549.17 (532)	−0.45 (−0.55 to −0.35)	−0.06 (−0.08 to −0.05)
Tropical Latin America	841.06 (520)	783.99 (363)	718.40 (359)	−1.14 (−1.19 to −1.08)	−0.05 (−0.05 to −0.05)
Western Europe	266.41 (79)	210.48 (53)	205.60 (53)	−1.27 (−1.42 to −1.12)	−0.02 (−0.03 to −0.01)
Western Sub-Saharan Africa	1602.99 (733)	3932.72 (708)	7060.90 (696)	−0.08 (−0.17 to 0.01)	−0.06 (−0.06 to −0.06)
High SDI	821.79 (98)	768.62 (71)	911.54 (71)	−0.92 (−1.04 to −0.79)	−0.03 (−0.04 to −0.02)
High–middle SDI	2925.49 (281)	1994.56 (158)	2335.54 (157)	−2.09 (−2.18 to −1.99)	−0.05 (−0.05 to −0.04)
Middle SDI	7977.35 (461)	7621.49 (326)	9855.23 (311)	−1.14 (−1.18 to −1.10)	−0.17 (−0.17 to −0.17)
Low–middle SDI	11,517.45 (975)	13,195.16 (702)	19,556.11 (675)	−1.06 (−1.11 to −1.00)	−0.14 (−0.14 to −0.13)
Low SDI	5143.75 (969)	8715.89 (757)	17,419.40 (723)	−0.81 (−0.84 to −0.78)	−0.13 (−0.14 to −0.12)

**Table 2:** The top three countries with the highest ASPR or ASDR for ID in 1990, 2021, and 2050. Abbreviations: ASPR, age-standardized prevalence rate; ASDR, age-standardized DALYs rate; ID, iron deficiency.

Year	ASPR (per 100,000)	ASDR (per 100,000)
1990		
	India (36,853)	Yemen (1440)
	Nepal (36,048)	Pakistan (1313)
	Pakistan (35,972)	Senegal (1269)
2021		
	Senegal (34,421)	Yemen (1405)
	Mali (34,233)	Mozambique (1149)
	Pakistan (33,942)	Mali (1093)
2050		
	Mali (35,070)	Yemen (1388)
	Senegal (34,132)	Mali (1181)
	Zambia (33,149)	Mozambique (1177)

## Data Availability

The datasets generated for this study can be found in the GBD at https://vizhub.healthdata.org/gbd-results/ (accessed on 1 August 2024).

## Supplementary Materials

The following supporting information can be downloaded at: https://www.mdpi.com/article/10.3390/nu16203434/s1, Figure S1: Evaluation of Model Predictive Performance for Predicting Prevalence and DALYs Rate of ID; Figure S2: Number and Rate of ID from 1990 to 2050 at the Global Level by Genders and Age Groups; Figure S3: Male-to-Female Ratio of Prevalence/DALYs Rate for ID in Different Age Groups; Figure S4: Age, Period, and Birth Cohort Effects for Global ID Prevalence and DALYs Rate; Figure S5: SHAP Summary Plot of Feature Contributions Ranked by Mean |SHAP| Values and SHAP Dependence Plots for Each Feature in the XGBoost Model Predicting ID Prevalence Rate in Senegal; Figure S6: SHAP Summary Plot of Feature Contributions Ranked by Mean |SHAP| Values and SHAP Dependence Plots for Each Feature in the XGBoost Model Predicting ID Prevalence Rate in Mali; Figure S7: SHAP Summary Plot of Feature Contributions Ranked by Mean |SHAP| Values and SHAP Dependence Plots for Each Feature in the XGBoost Model Predicting ID Prevalence Rate in Pakistan; Figure S8: SHAP Summary Plot of Feature Contributions Ranked by Mean |SHAP| Values and SHAP Dependence Plots for Each Feature in the XGBoost Model Predicting ID DALYs Rate in Yemen; Figure S9: SHAP Summary Plot of Feature Contributions Ranked by Mean |SHAP| Values and SHAP Dependence Plots for Each Feature in the XGBoost Model Predicting ID DALYs Rate in Mozambique; Figure S10: SHAP Summary Plot of Feature Contributions Ranked by Mean |SHAP| Values and SHAP Dependence Plots for Each Feature in the XGBoost Model Predicting ID DALYs Rate in Mali; Table S1: All Ages Prevalent Cases, DALYs, Prevalence Rate, and DALYs Rate by Nation/Region, Year, and Sex. Abbreviations: DALYs, disability-adjusted life years; SDI, Socio-Demographic Index; UI, uncertainty interval; Table S2: ASPR and ASDR by Nation/Region, Year, and Sex. Abbreviations: ASPR, age-standardized prevalence rate; ASDR, age-standardized DALYs rate; SDI, Socio-Demographic Index; UI, uncertainty interval; Table S3: EAPCs of ASR from 1990 to 2021 and from 2021 to 2050 at the National or Regional Level. Abbreviations: EAPC, estimated annual percentage change; ASRs, age-standardized rates; SDI, Socio-Demographic Index; CI, confidence interval; Table S4: Prevalence and DALYs Rates by Nation/Region, Year, Sex, and Age Group. Abbreviations: DALYs, disability-adjusted life years; SDI, Socio-Demographic Index; UI, uncertainty interval; Table S5: Age Effect on Prevalence and DALYs Rates. Abbreviations: DALYs, disability-adjusted life years; CI, confidence interval; Table S6: Period Effect on Prevalence and DALYs Rates. Abbreviations: DALYs, disability-adjusted life years; RR, rate ratio; CI, confidence interval; Table S7: Cohort Effect on Prevalence and DALYs Rates. Abbreviations: DALYs, disability-adjusted life years; RR, rate ratio; CI, confidence interval; Video S1: ASPR of ID at the National Level from 1990 to 2050; Video S2: ASDR of ID at the National Level from 1990 to 2050.
