# Supplementary material for: Iron Deficiency: Global Trends and Projections from 1990 to 2050
Source: Nutrients. 2024 Oct 10;16(20):3434. doi: 10.3390/nu16203434 (PMC11510637; doi:10.3390/nu16203434)
Supplement: Supplementary file 1 [file nutrients-16-03434-s001.zip › nutrients-3241132-supplementary files.pdf]

TABLE OF CONTENTS

Supplementary Materials File S1. Calculation of EAPC 2
Supplementary Materials File S2. APC Modelling Analysis of Age, Period and Birth Cohort Effects on Global ID Prevalence and DALYs 3
Supplementary Materials File S3. Explorer Optimal Hyperparameters through Grid Search and Cross Validation 4
Figure S1. Evaluation of Model Predictive Performance for Predicting Prevalence and DALYs Rate of ID 5
Figure S2. Number and Rate of ID from 1990 to 2050 at the Global Level by Genders and Age Groups 5
Figure S3. Male-to-Female Ratio of Prevalence/DALYs Rate for ID in Different Age Groups 6
Figure S4. Age, Period, and Birth Cohort Effects for Global ID Prevalence and DALYs Rate 7
Figure S5. SHAP Summary Plot of Feature Contributions Ranked by Mean |SHAP| Values and SHAP Dependence Plots for Each Feature in the XGBoost Model Predicting ID Prevalence Rate in Senegal 8
Figure S6. SHAP Summary Plot of Feature Contributions Ranked by Mean |SHAP| Values and SHAP Dependence Plots for Each Feature in the XGBoost Model Predicting ID Prevalence Rate in Mali 9
Figure S7. SHAP Summary Plot of Feature Contributions Ranked by Mean |SHAP| Values and SHAP Dependence Plots for Each Feature in the XGBoost Model Predicting ID Prevalence Rate in Pakistan 10
Figure S8. SHAP Summary Plot of Feature Contributions Ranked by Mean |SHAP| Values and SHAP Dependence Plots for Each Feature in the XGBoost Model Predicting ID DALYs Rate in Yemen 11
Figure S9. SHAP Summary Plot of Feature Contributions Ranked by Mean |SHAP| Values and SHAP Dependence Plots for Each Feature in the XGBoost Model Predicting ID DALYs Rate in Mozambique 12
Figure S10. SHAP Summary Plot of Feature Contributions Ranked by Mean |SHAP| Values and SHAP Dependence Plots for Each Feature in the XGBoost Model Predicting ID DALYs Rate in Mali 13
Video S1. ASPR of ID at the National Level from 1990 to 2050 (See “Movie S1.mp4” in the Supplementary ZIP) 14
Video S2. ASDR of ID at the National Level from 1990 to 2050 (See “Movie S2.mp4” in the Supplementary ZIP). 14

Supplementary Materials File S1. Calculation of EAPC

EAPCs were calculated using the follow regression model:

$$y = \beta_0 + \beta_1 * x_1$$

Where x_1 is the calendar year, y is the natural logarithm of the ASR, which could represent different rates such as the ASPR or ASDR. β_0 is the intercept, and β_1 is the coefficient related to the calendar year.

EAPCs, which indicate the trends in ASRs over specified time frames (e.g., 1990-2021, or 2021-2050), were derived using the formula[1]:

$$EAPC = 100 * (exp(\beta_1) - 1)$$

The confidence intervals for the EAPCs were determined by applying the upper and lower limits of β_1 from the regression model.

Abbreviations:

EAPC: estimated annual percentage change

ASR: age-standardized rate

ASPR: age-standardized prevalence rate

ASDR: age-standardized DALYs rate

Reference

1. Hankey BF, Ries LA, Kosary CL, Feuer EJ, Merrill RM, Clegg LX, et al. Partitioning linear trends in age-adjusted rates. Cancer causes & control : CCC. 2000 Jan;11(1):31-5. PMID: 10680727. doi: 10.1023/a:1008953201688.

Supplementary Materials File S2. APC Modelling Analysis of Age, Period and Birth Cohort Effects on Global ID Prevalence and DALYs

We used GBD 2021 data on global prevalence/DALYs rates and population counts as inputs for our APC model. To address the identification challenge due to the inherent collinearity between age, period, and cohort, we employed the IE method, which improves the accuracy and robustness of the model parameters[1]. The model requires equal intervals for both age and period categories. Accordingly, we grouped the prevalence/DALYs rates, and population data for individuals aged 0 to 85+ into 18 age brackets, each spanning 5 years (e.g., <5, 5-9, ..., 85+).

For the analysis covering the years 1990 to 2049, the data were segmented into 12 time periods (e.g., 1990-1994, 1995-1999, ..., 2045-2049). Birth cohorts were divided into 29 groups, ranging from 1903-1907 (with a midpoint of 1905) to 2043-2047 (midpoint 2045). Population, prevalent cases, and DALYs for each period and birth cohort were calculated as averages over these intervals. We used Rate Ratios (RRs) to assess the effects of period and cohort on ID outcomes. An RR greater than 1 indicates a higher risk compared to the reference group, while an RR less than 1 suggests a lower risk.

Abbreviations:

APC: Age-period-cohort
ID: iron deficiency
DALYs: disability-adjusted life years
GBD: Global Burden of Disease
IE: intrinsic Estimator
RR: rate ratio

Reference

1. Yang, Yang., Sam, Schulhofer-Wohl., Wenjiang, J., Fu., Kenneth, C., Land. (2008). The intrinsic estimator for age-period-cohort analysis: What it is and how to use it. American Journal of Sociology, 113(6):1697-1736. doi: 10.1086/587154

Supplementary Materials File S3. Explorer Optimal Hyperparameters through Grid Search and Cross Validation

To improve model performance and prediction accuracy, we performed grid search to optimize key hyperparameters such as *eta* (learning rate), *max_depth* (maximum tree depth), and *nrounds* (number of boosting rounds). We applied 5-fold cross-validation, using root mean square error (RMSE) to evaluate each combination of hyperparameters.

The *max_depth* parameter governs how complex the trees in the model can be. Deeper trees allow the model to identify intricate patterns, but they may also lead to overfitting. In contrast, shallower trees may generalize better to new data but can miss subtleties within the dataset. We tested *max_depth* values between 5 and 12 to examine how tree complexity affects model performance.

The *eta* parameter adjusts the step size during the model's optimization process. A smaller *eta* encourages more careful learning and often requires more iterations to converge, while a larger eta speeds up the process but risks converging to a less optimal solution. We experimented with specific *eta* values: 0.01, 0.015, 0.025, 0.05, 0.1, 0.15, 0.2, 0.25, and 0.3, to carefully examine the impact of learning rate adjustments.

The *nrounds* parameter controls how many iterations of boosting are applied during training. Too few iterations can result in underfitting, while too many can lead to overfitting. We tested *nrounds* from 100 to 1500 in steps of 100 to assess the balance between model capacity and iteration count.

By systematically adjusting these hyperparameters, we aimed to identify the best combination to enhance model performance and predictive accuracy.

Abbreviations:

RMSE: root mean square error

Figure S1. Evaluation of Model Predictive Performance for Predicting Prevalence and DALYs Rate of ID

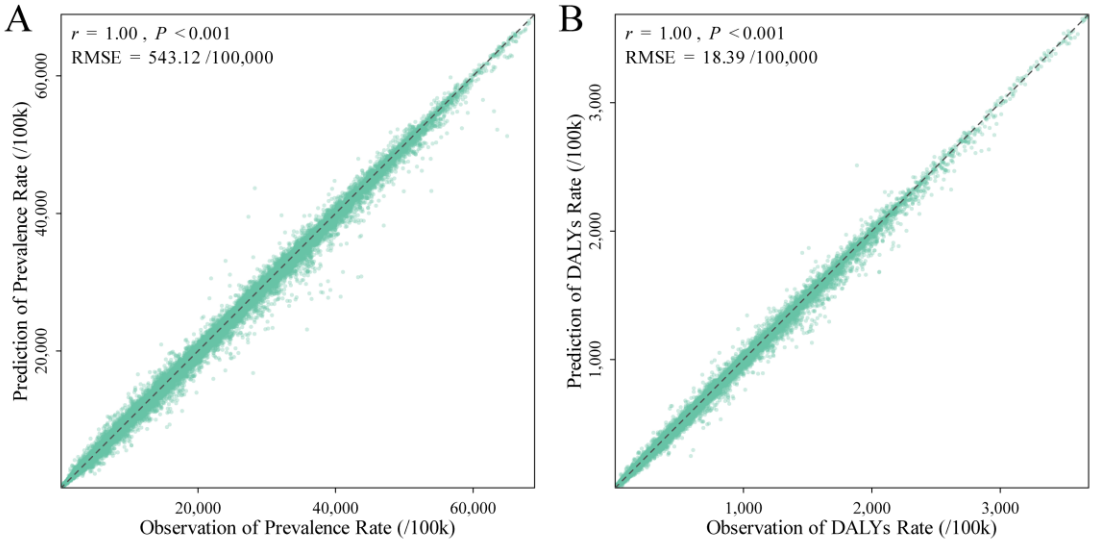

Abbreviations:

DALYs: disability-adjusted life years

ID: iron deficiency

Figure S2. Number and Rate of ID from 1990 to 2050 at the Global Level by Genders and Age Groups

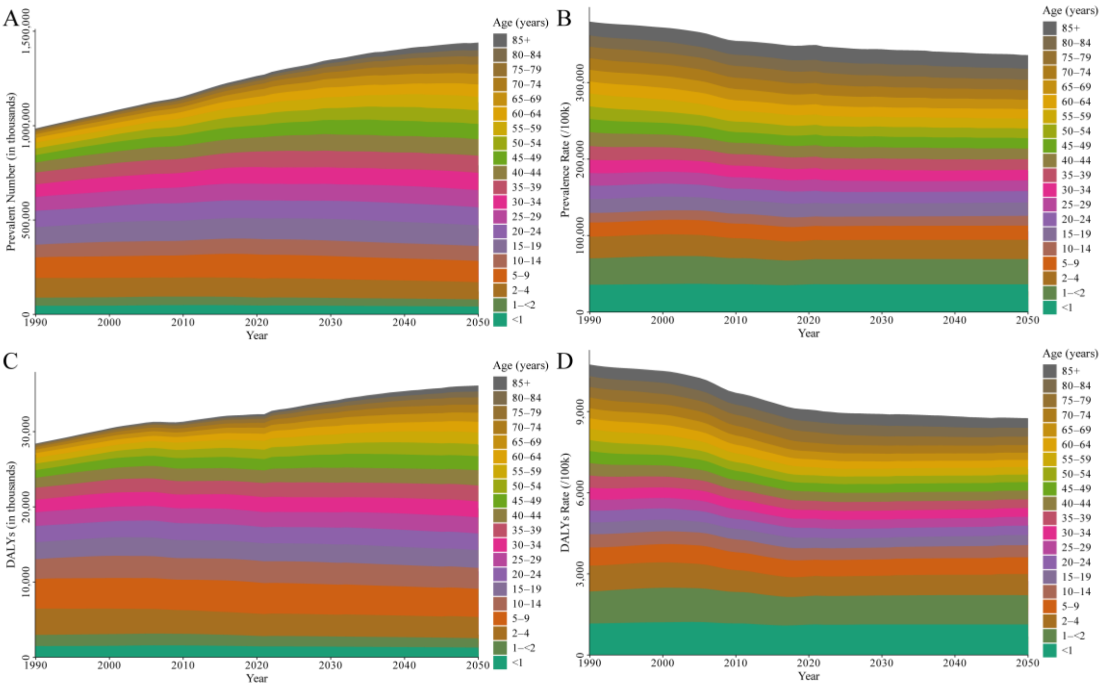

(A) Prevalent case; (B) Prevalence rate; (C) DALYs; (D) DALYs rate

Abbreviations:

ID, iron deficiency

DALYs, disability-adjusted life years

Figure S3. Male-to-Female Ratio of Prevalence/DALYs Rate for ID in Different Age Groups

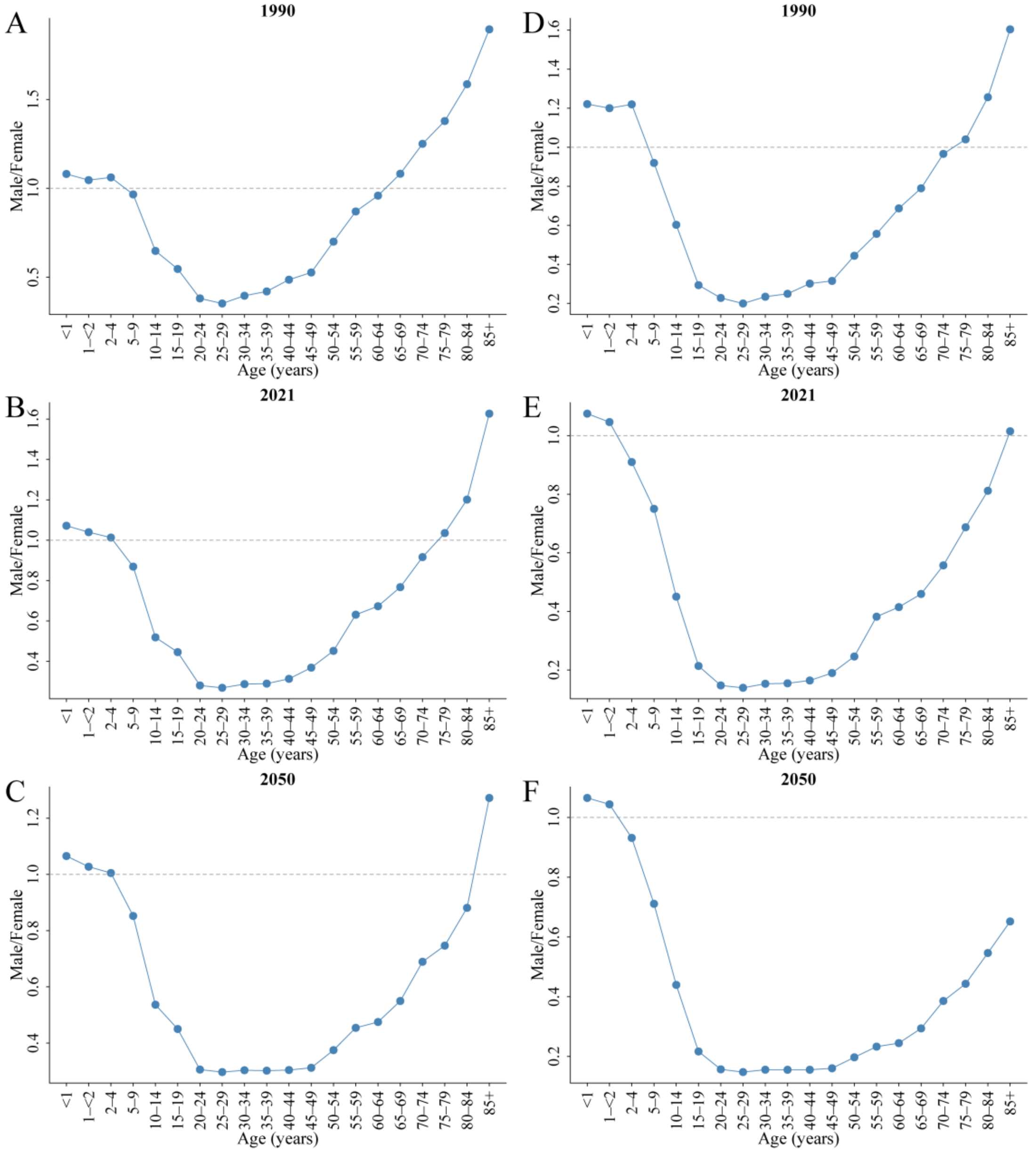

(A-C) Prevalence rate in 1990 (A), 2021 (B), and 2050 (C); (D-F) DALYs rate in 1990 (D), 2021 (E), and 2050 (F).

Abbreviations:

DALYs, disability-adjusted life years

ID, iron deficiency

Figure S4. Age, Period, and Birth Cohort Effects for Global ID Prevalence and DALYs Rate

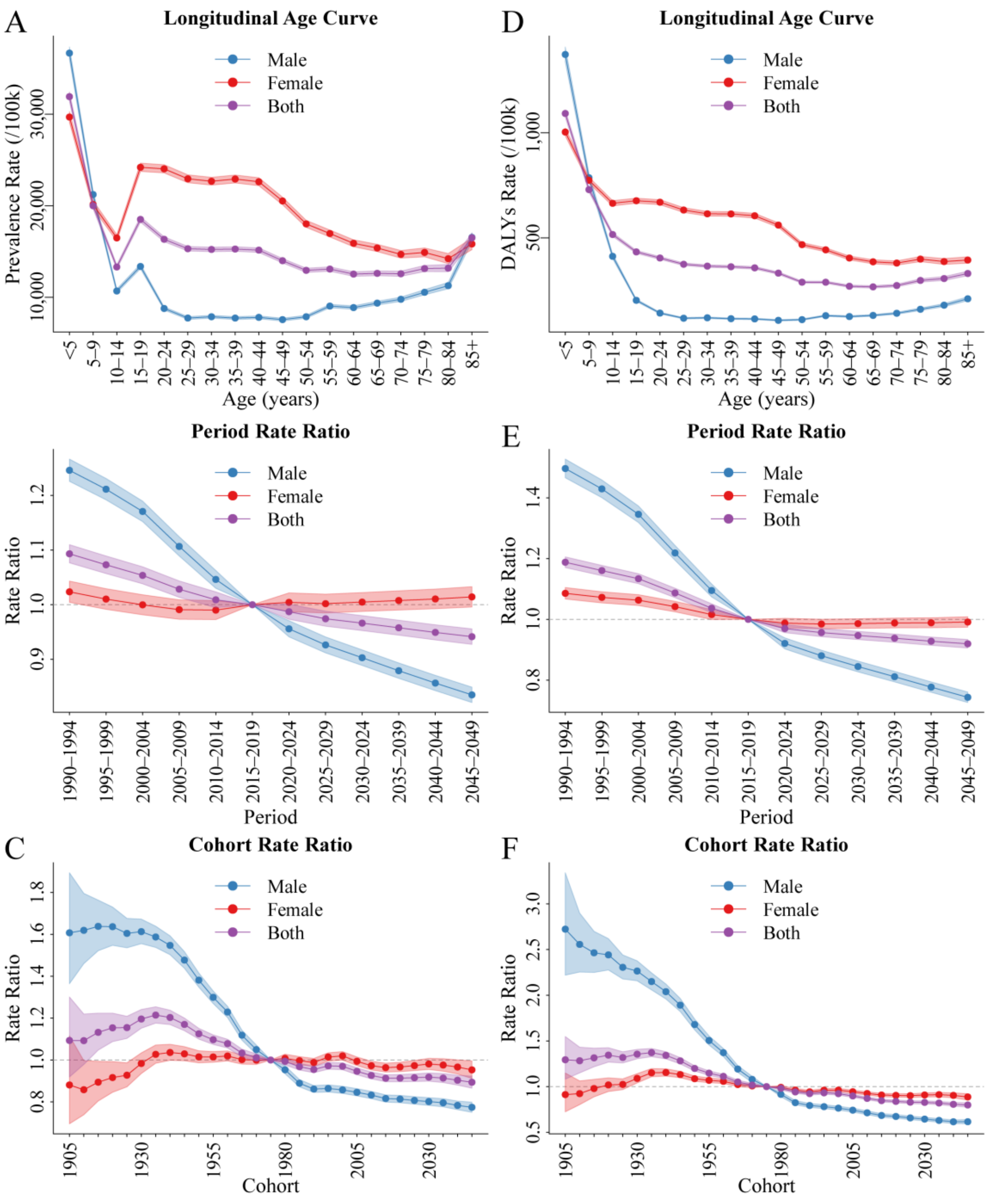

(A-C) The age (A), period (B), and cohort (C) effects of prevalence. (D-F) The age (D), period (E), and cohort (F) effects of DALYs rate.

Abbreviation:

ID, iron deficiency

DALYs, disability-adjusted life years

Figure S5. SHAP Summary Plot of Feature Contributions Ranked by Mean |SHAP| Values and SHAP Dependence Plots for Each Feature in the XGBoost Model Predicting ID Prevalence Rate in Senegal

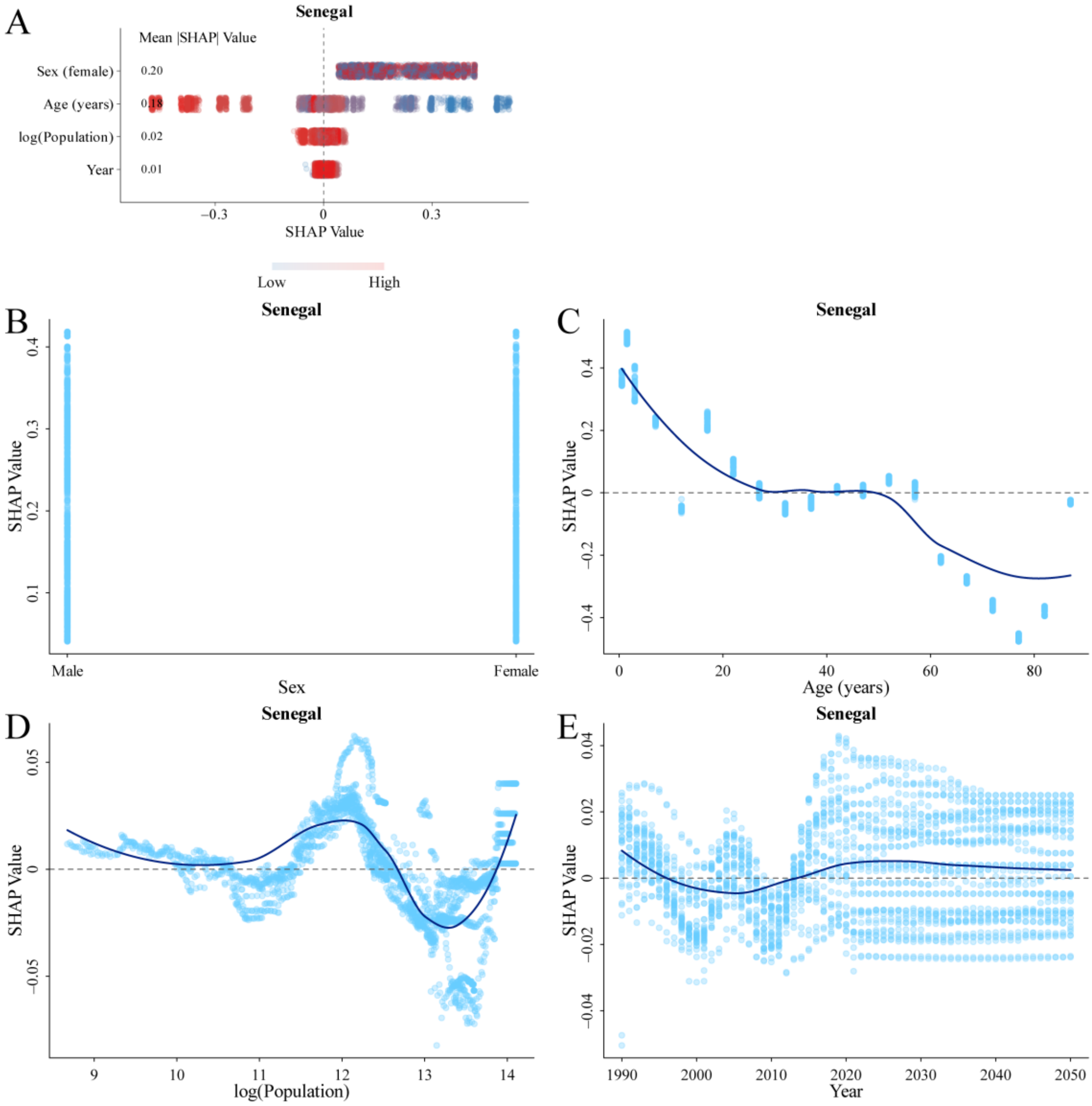

(A) Summary plot, (B-E) The dependence plot showing the contribution of gender (B), age (C), log (population) (D), and year (E).

Abbreviations:

SHAP, SHapley Additive exPlanations

ID, iron deficiency

Figure S6. SHAP Summary Plot of Feature Contributions Ranked by Mean |SHAP| Values and SHAP Dependence Plots for Each Feature in the XGBoost Model Predicting ID Prevalence Rate in Mali

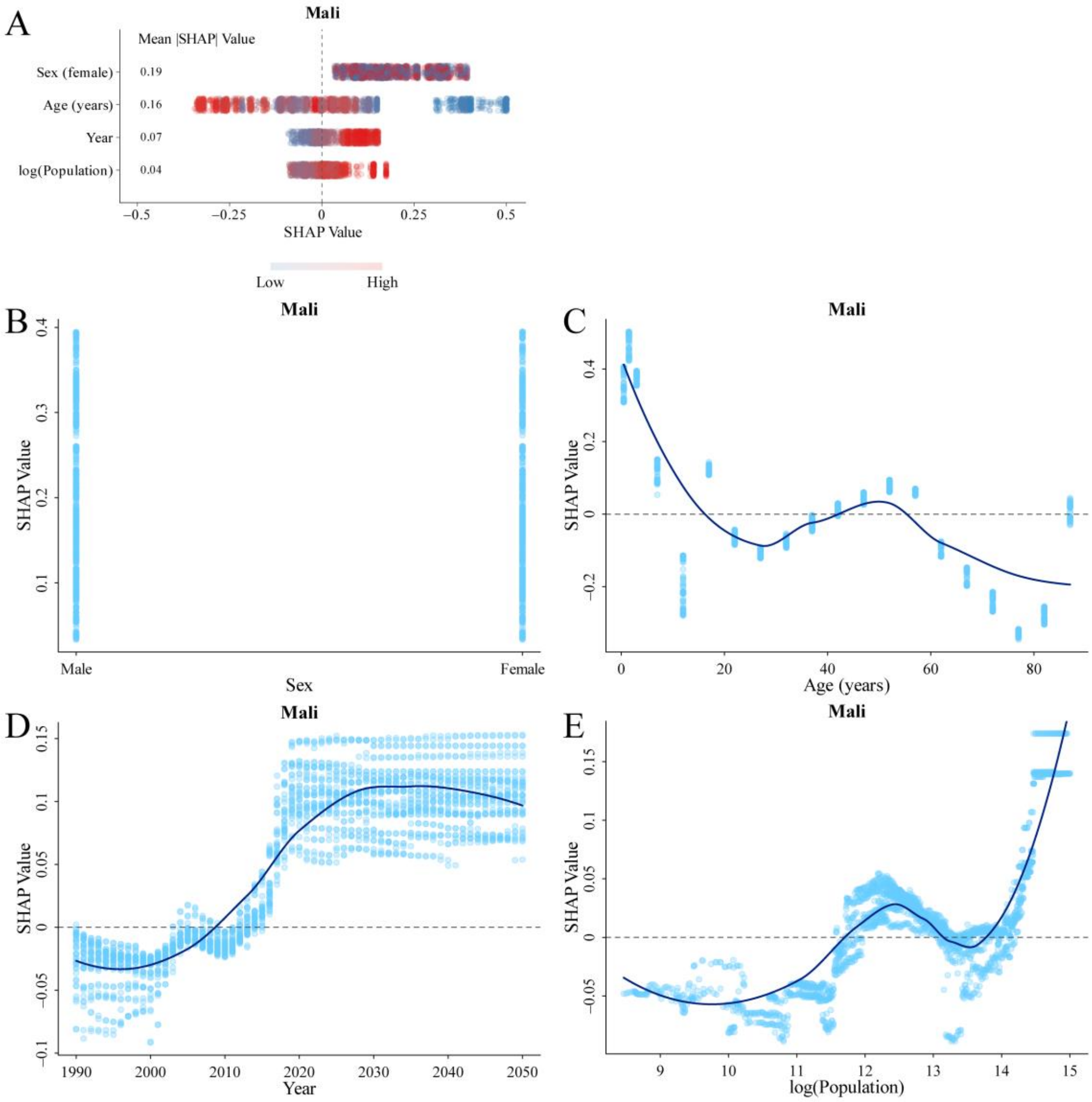

(A) Summary plot, (B-E) The dependence plot showing the contribution of gender (B), age (C), year (D), and log (population) (E).

Abbreviations:

SHAP, SHapley Additive exPlanations

ID, iron deficiency

Figure S7. SHAP Summary Plot of Feature Contributions Ranked by Mean |SHAP| Values and SHAP Dependence Plots for Each Feature in the XGBoost Model Predicting ID Prevalence Rate in Pakistan

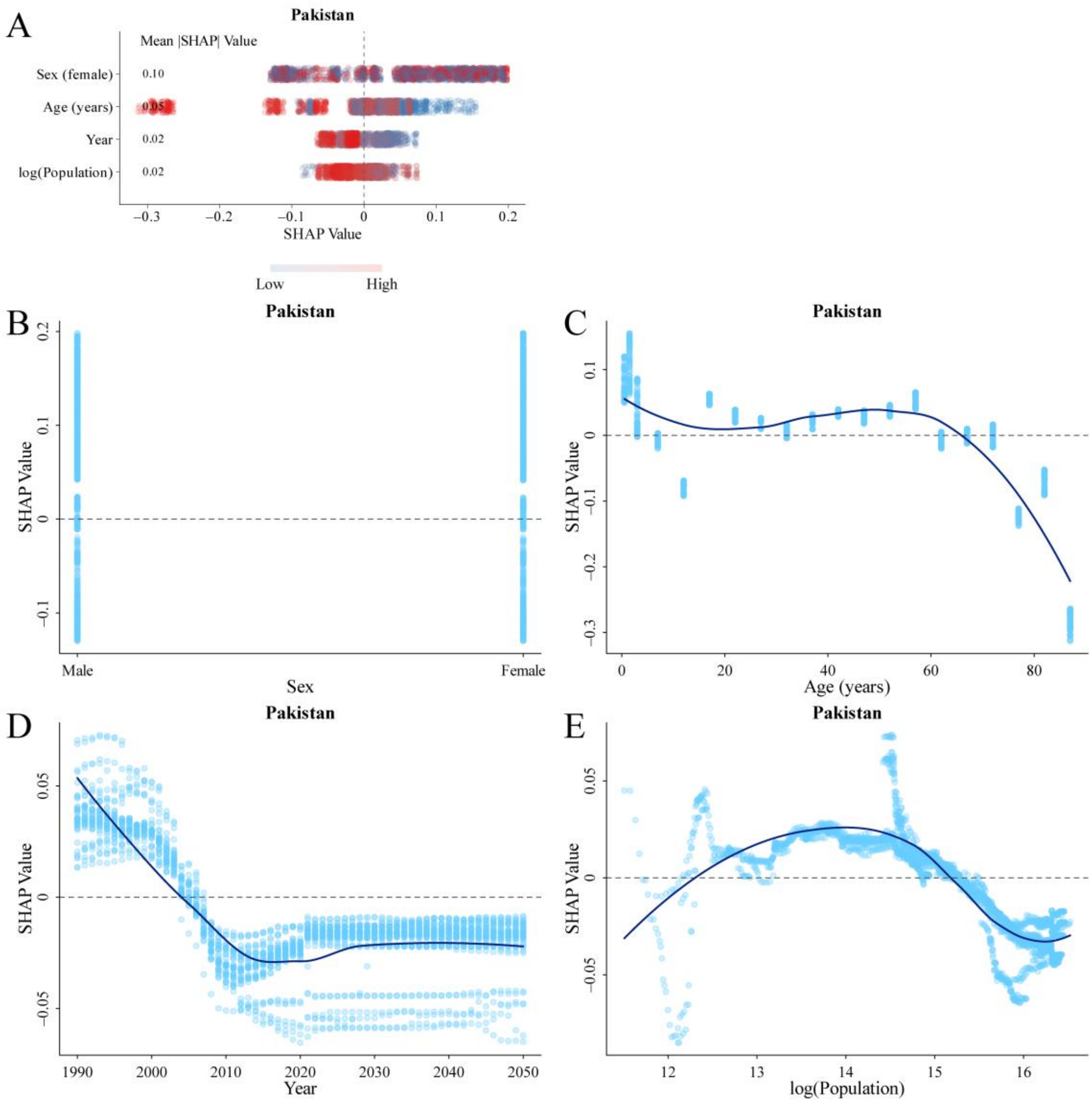

(A) Summary plot, (B-E) The dependence plot showing the contribution of gender (B), age (C), year (D), and log (population) (E).

Abbreviations:

SHAP, SHapley Additive exPlanations

ID, iron deficiency

Figure S8. SHAP Summary Plot of Feature Contributions Ranked by Mean |SHAP| Values and SHAP Dependence Plots for Each Feature in the XGBoost Model Predicting ID DALYs Rate in Yemen

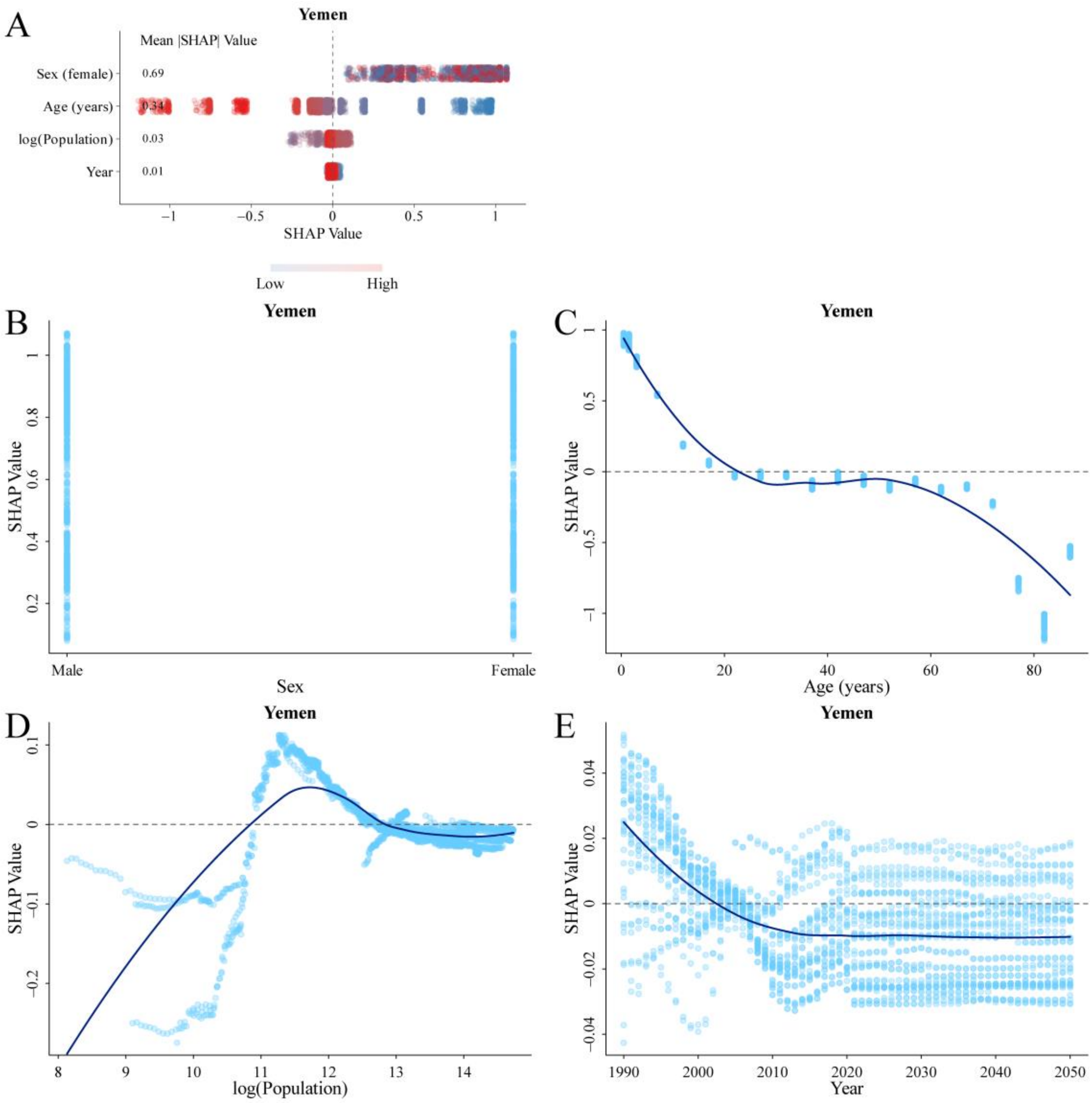

(A) Summary plot, (B-E) The dependence plot showing the contribution of gender (B), age (C), log (population) (D), and year (E).

Abbreviations:

SHAP, SHapley Additive exPlanations

ID, iron deficiency

DALYs, disability-adjusted life years

Figure S9. SHAP Summary Plot of Feature Contributions Ranked by Mean |SHAP| Values and SHAP Dependence Plots for Each Feature in the XGBoost Model Predicting ID DALYs Rate in Mozambique

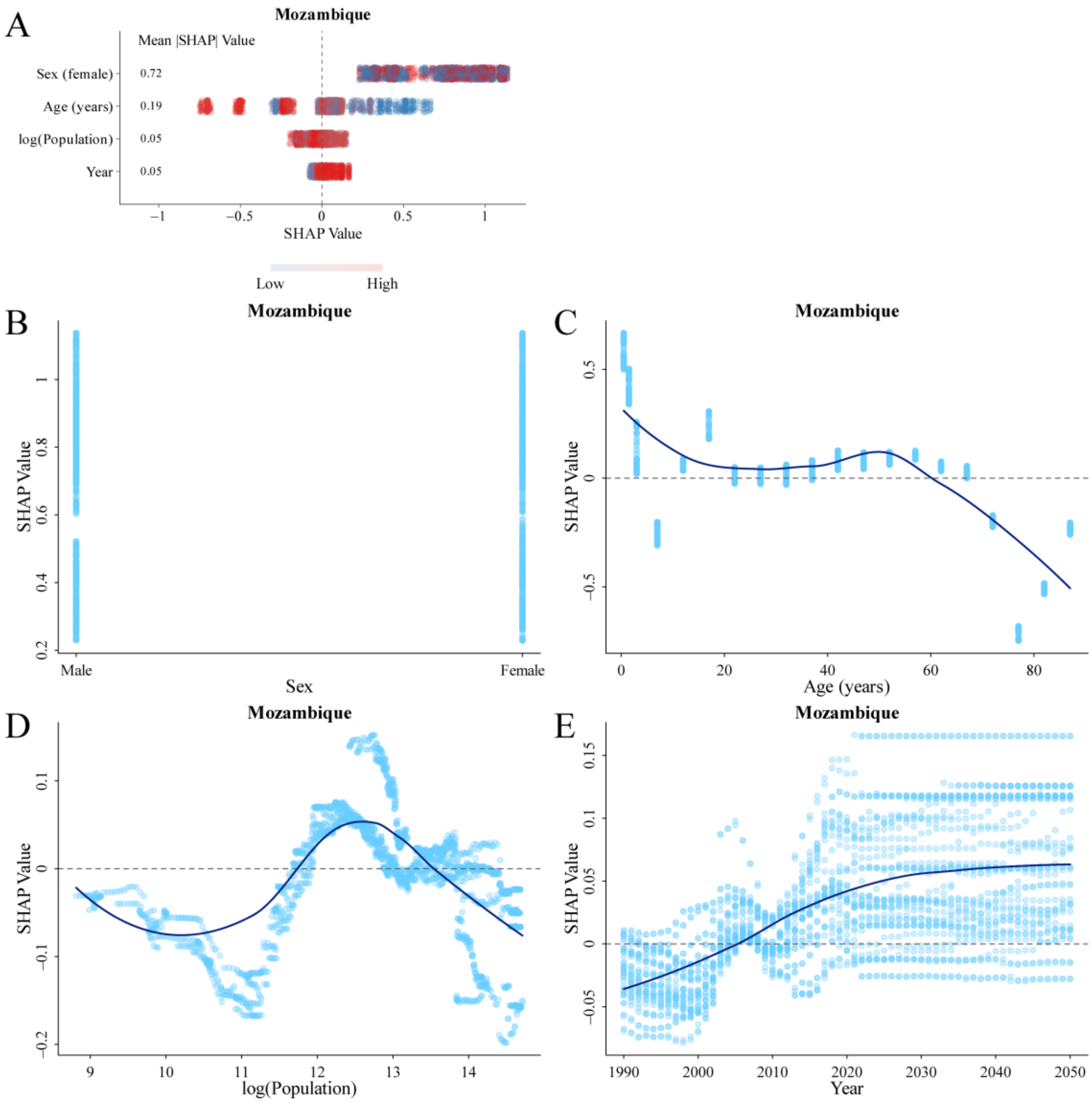

(A) Summary plot, (B-E) The dependence plot showing the contribution of gender (B), age (C), log (population) (D), and year (E).

Abbreviations:

SHAP, SHapley Additive exPlanations

ID, iron deficiency

DALYs, disability-adjusted life years

Figure S10. SHAP Summary Plot of Feature Contributions Ranked by Mean |SHAP| Values and SHAP Dependence Plots for Each Feature in the XGBoost Model Predicting ID DALYs Rate in Mali

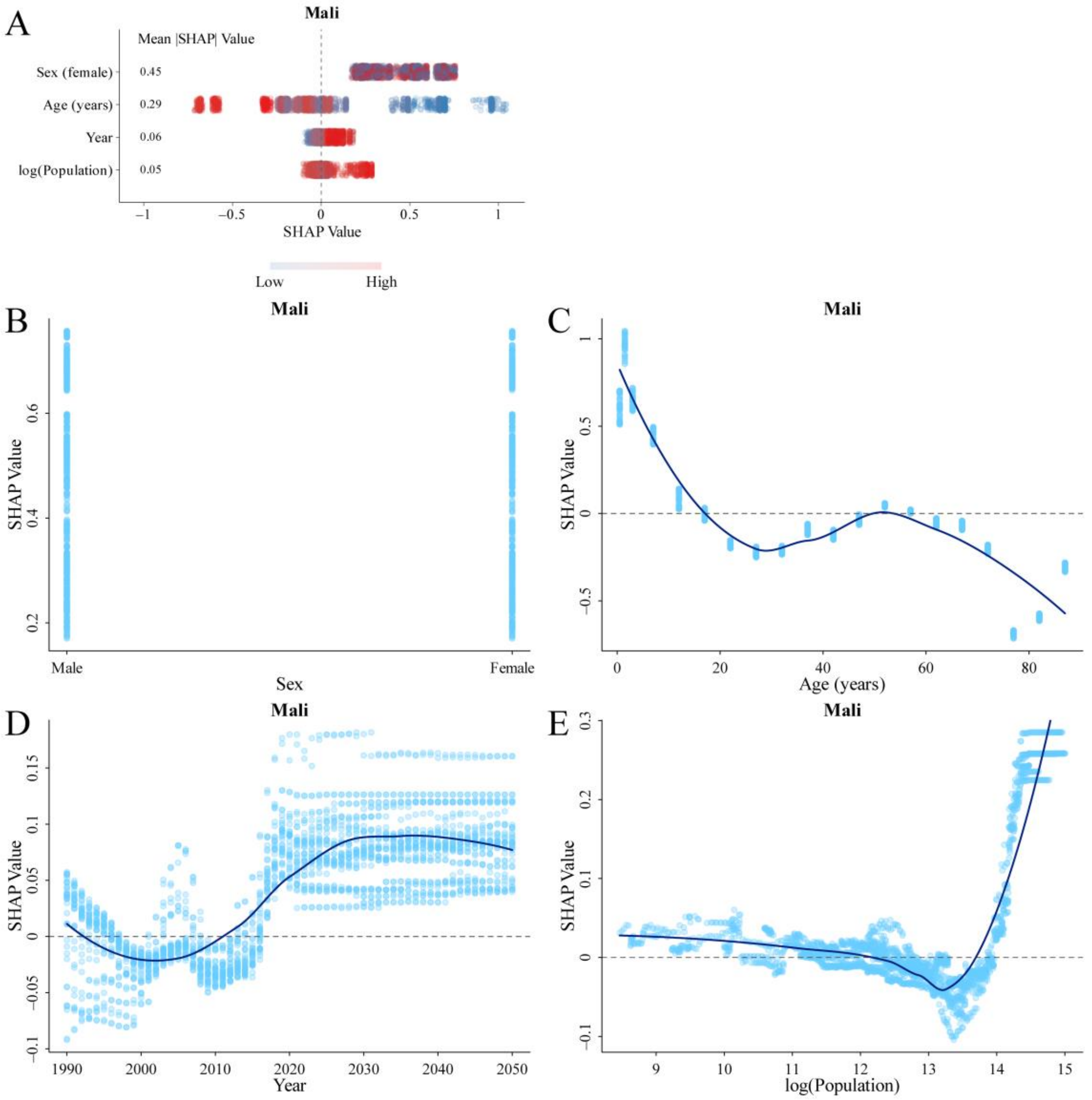

(A) Summary plot, (B-E) The dependence plot showing the contribution of gender (B), age (C), year (D), and log (population) (E).

Abbreviations:

SHAP, SHapley Additive exPlanations

ID, iron deficiency

DALYs, disability-adjusted life years

Video S1. ASPR of ID at the National Level from 1990 to 2050 (See “Movie S1.mp4” in the Supplementary ZIP)

Abbreviations:
ASPR, age-standardized prevalence rate
ID, iron deficiency

Video S2. ASDR of ID at the National Level from 1990 to 2050 (See “Movie S2.mp4” in the Supplementary ZIP).

Abbreviations:
ASDR, age-standardized DALYs rate
ID, iron deficiency
